# Metamorphosis of Twin Screw Extruder-Based Granulation Technology: Applications Focusing on Its Impact on Conventional Granulation Technology

**DOI:** 10.1208/s12249-021-02173-w

**Published:** 2021-12-14

**Authors:** Rajat Radhakrishna Rao, Abhijeet Pandey, Aswathi R. Hegde, Vijay Induvadan Kulkarni, Chetan Chincholi, Vinay Rao, Indu Bhushan, Srinivas Mutalik

**Affiliations:** 1 Department of Pharmaceutics, Manipal College of Pharmaceutical Sciences, Manipal Academy of Higher Education, Manipal, 576104 Karnataka State India; 2 STEERLife, Steer Engineering Pvt Ltd, No. 290, 4th Main Road, Ganapathy Nagar, Phase 3, Peenya Industrial Area, Peenya, Bangalore, 560058 Karnataka State India

**Keywords:** Continuous manufacturing, Twin screw granulation, Extruder design, Hot melt extruder, Twin screw extrusion, Process analytical techniques

## Abstract

In order to be at pace with the market requirements of solid dosage forms and regulatory standards, a transformation towards systematic processing using continuous manufacturing (CM) and automated model-based control is being thought through for its fundamental advantages over conventional batch manufacturing. CM eliminates the key gaps through the integration of various processes while preserving quality attributes via the use of process analytical technology (PAT). The twin screw extruder (TSE) is one such equipment adopted by the pharmaceutical industry as a substitute for the traditional batch granulation process. Various types of granulation techniques using twin screw extrusion technology have been explored in the article. Furthermore, individual components of a TSE and their conjugation with PAT tools and the advancements and applications in the field of nutraceuticals and nanotechnology have also been discussed. Thus, the future of granulation lies on the shoulders of continuous TSE, where it can be coupled with computational mathematical studies to mitigate its complications.

## INTRODUCTION

Pharmaceutical manufacturing has been carried out using batch processes since its inception, which, while tested and proved, are inept and financially exorbitant. These last several years, with the growing demand for solid dosage forms and expiring patents of drug products and molecules, acceleration, and de-risking of process and product development is of primal importance. Considering this, a change towards continuous manufacturing (CM) by the pharmaceutical industry and the regulatory bodies has drawn considerable attention by virtue of its inherent advantages towards process development, smaller physical footprint, manufacturing flexibility, product quality control, and overall expenditure to develop novel processes or by using familiar machinery in a non-traditional way (1–3). Continuous manufacturing (CM) is an up-and-coming technology in the pharma world that offers several benefits over the traditional batch processes, including production rate litheness, quality, robustness, and cost improvements (4, 5).

**Fig. 1:**
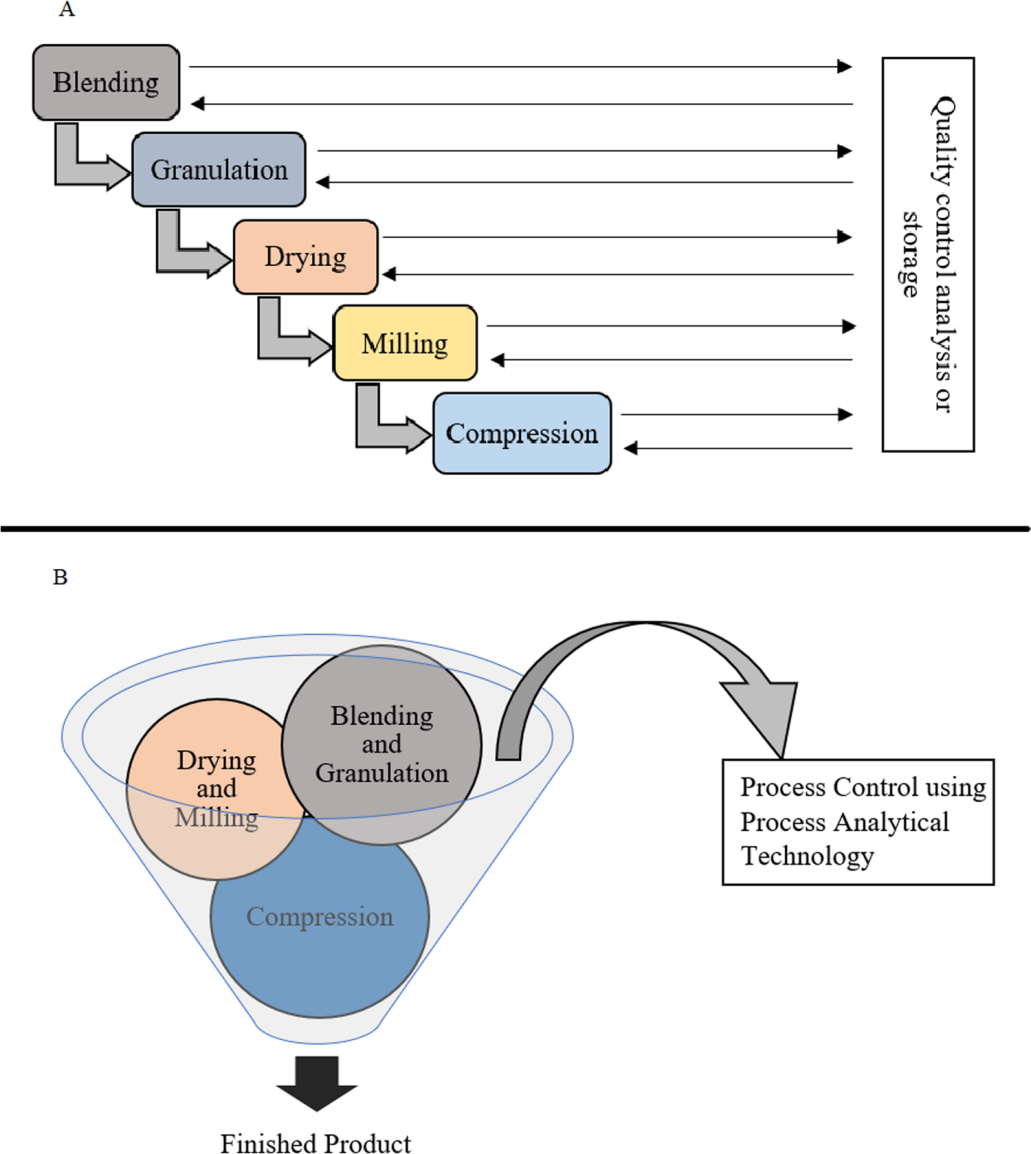
Comparing batch manufacturing *vs* continuous manufacturing

In a traditional solid drug product manufacturing process (Fig. 1A) that involves multiple processing steps (dispensing, mixing, high shear granulation, drying, milling, tableting, etc.), the FDA oversees each unit operation during manufacturing to ensure that the drug product supply is of high quality consistently. Previously, a lack of versatility and reproducibility caused many reservations in the pharmaceutical manufacturing industries, resulting in subpar drug products. This meant excessive additional work for the FDA so as to regulate practically every aspect of a certain manufacturing process (6). To keep up with the current market requirements and regulatory standards, most industries have initiated inculcating continuous manufacturing, a systematic approach to designing processes, and automated model-based control. This strategy has contributed to the immense quality and consistency improvements (7). A well-modeled continuous manufacturing system integrates multiple activities into a single production run (Fig. 1B) without compromising the quality by continuous tracking of critical process parameters (CPPs) and critical quality attributes (CQAs) via tests in the process flow using process analytical technology (PAT). This helps in the detection of variations in real time and corrects them to keep the process and the CQAs of the finished product within the standard limits. CM avoids the compulsion for managing process intermediates and helps with reducing the process cycle and finished product timeline (8, 9).

One of the major bottlenecks of the pharmaceutical industry is the development pace and an endless number of trials just to chart design space to work with. CM eliminates multiple development scales as teams move from lab scale to pilot scale through to large-scale establishment seamlessly (10). Extrusion technology has been heavily investigated in the pharmaceutical industry for the ability to carry out continuous manufacturing of various pharmaceutical formulations and scalability and for its potential as a possible alternative to the conventional route (11).

## Extruder Types

**Fig. 2:**
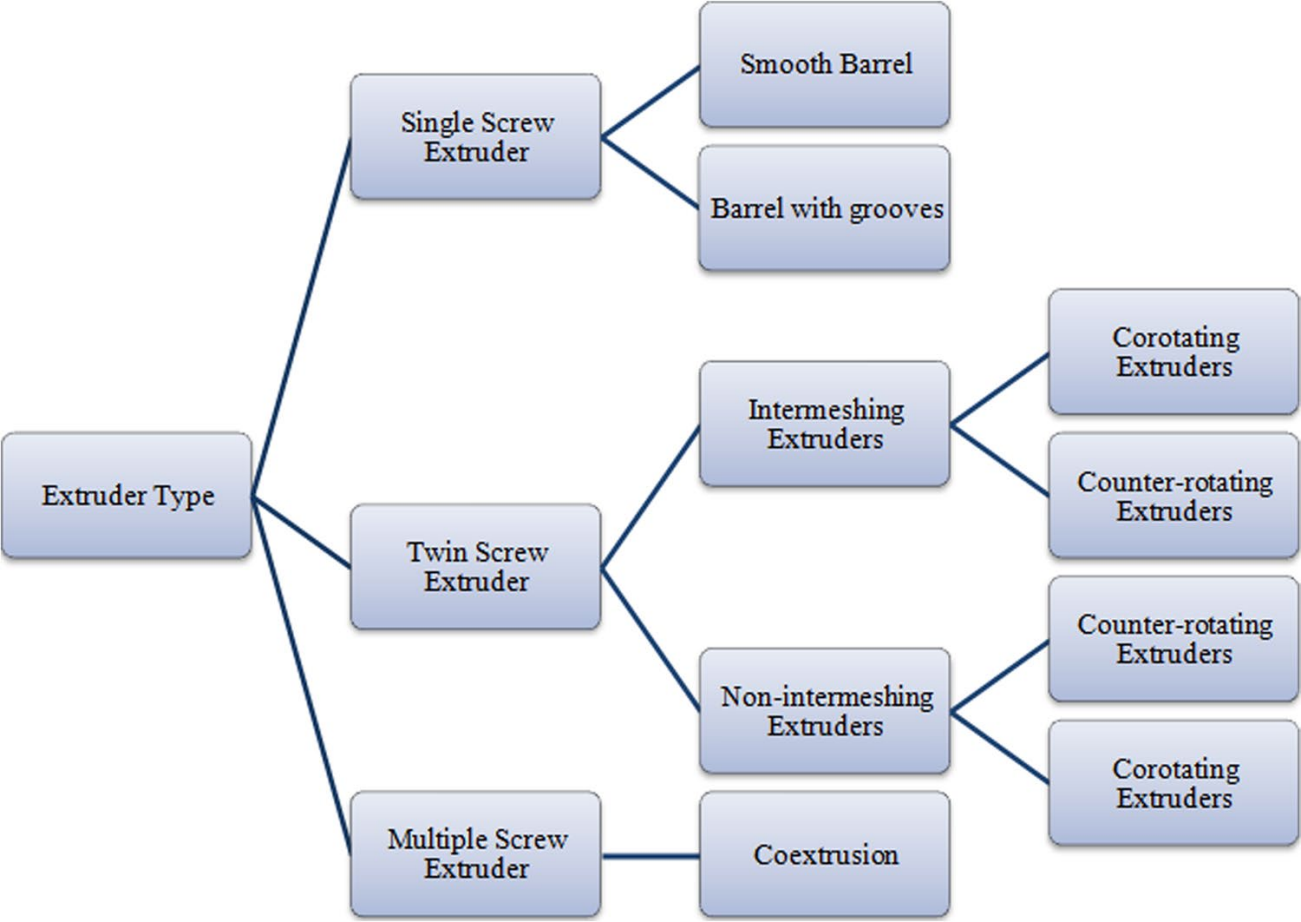
Categories of extruders

Depending on the number of shafts and/or screws present, extruder variations can be categorized as single screw extruders, twin screw extruders, and multiple screw extruders. Figure 2 describes the categorization of extruders based on shaft/screws.

### Single Screw Extruder

With a smooth barrel, as well as those that include grooves and/or pins, single screw extruders are used largely for melting and pressure buildup in plastic processing. To achieve this, combination of frictional forces in the solids conveying zone and viscous forces in the melt transport zone is required. As a result, the single screw extrusion process is strongly reliant on the frictional and viscous characteristics of the material (12).

### Twin Screw Extruder (TSE)

As single screw extruders have limited mixing capabilities, twin screws (with two shafts) are frequently used to overcome this limitation. Twin screw extruders, as the name implies, typically use two side-by-side screws (13). During pharmaceutical processing of API-polymer blends, TSEs have proved to be more consistent and dominant over their single screw counterparts. The TSE is a process of forcing raw materials into an extruder with screw elements at elevated temperatures and induced shear to plasticize the components within the machine to ultimately produce extrudates, thus obtaining the desired product (14). TSEs can be configured to convey positively with help of a variety of configurations. The lesser the gap between screw turns, the tighter the seal and hence, the more positive the conveying feature. The frictional and viscous properties of the material have little impact on the conveying feature of the extruder.

**Fig. 3:**
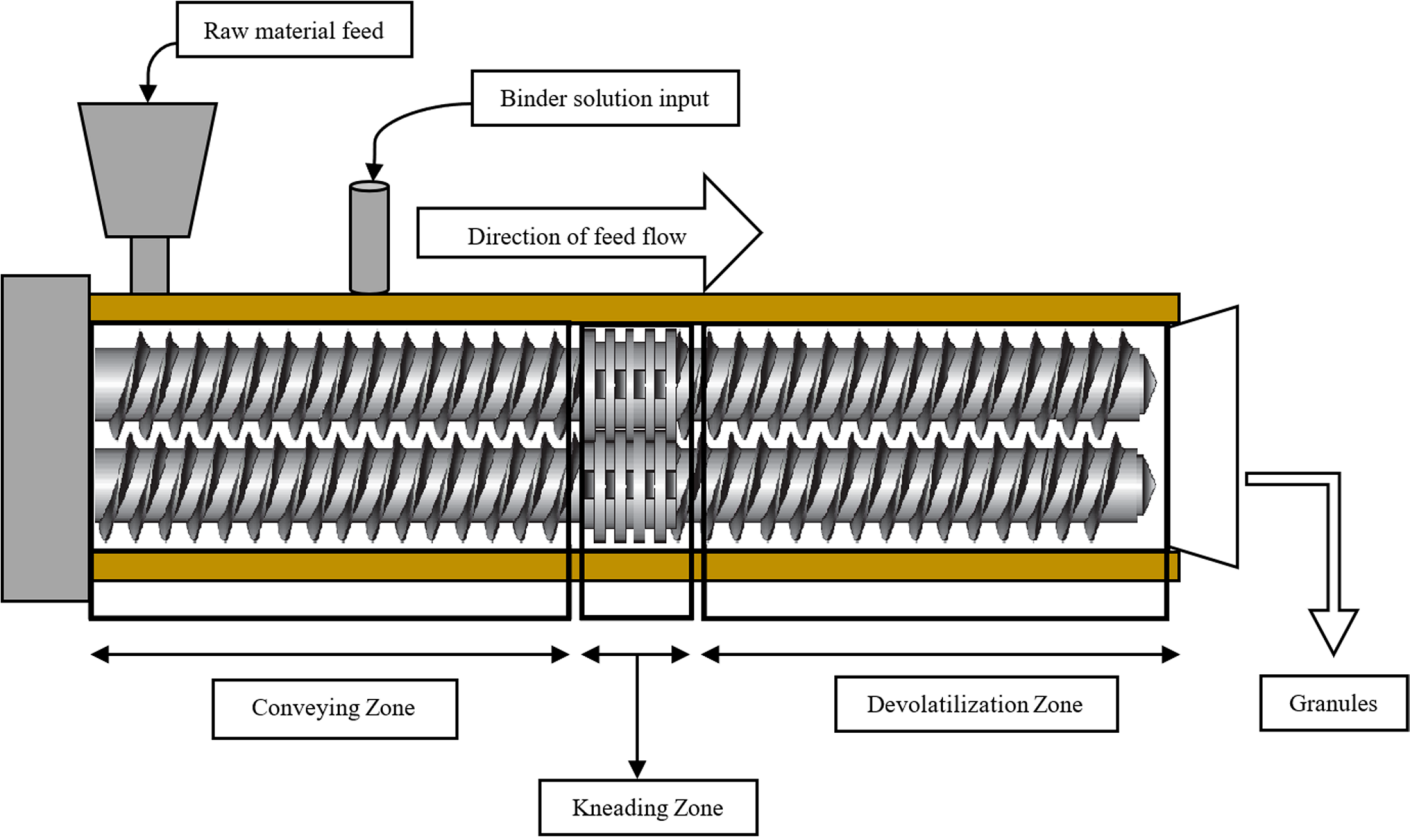
Material flow in a twin screw extruder (TSE)

The use of two screws enables the creation of a variety of different configurations and sets distinct conditions on all zones of the extruder, from material transfer from the hopper to the screws at kneading and conveying zone. The shafts of a twin screw extruder can spin in the same direction (co-rotating extruder) or in the opposite direction (counter-rotating extruder). The counter-rotating designs are most often used where high shear areas are desired, as the material is pushed through the space between the two screws when they are in contact. The extruder design also works well for dispersing particles. However, counter-rotating twin screw extruders have limited maximum screw speeds and output with potential air entrapment and high pressure generation. However, intermeshing twin screw extruders are self-wiping. They are the most essential industrial extruders because they can produce huge outputs while retaining superior mixing and conveying capabilities (15, 16). The first product that was approved by the USFDA way back in 1997 was Resulin® that utilized melt extrusion technology for improved bioavailability. Over the years, as a result of the untiring efforts of many researchers, twin screw extrusion-related activities accelerated and became the main focus of the pharmaceutical manufacturing sector. Twin screw granulation (TSG) is an approach using TSE for the transformation from batch processing of granulation technology to CM in the pharmaceutical industry (17). Though this technique has already been cited for pharmaceutical applications decades ago by Lindberg et al. (1988) and Gamlen and Eardley (1986) for effervescent and paracetamol granules, respectively (18–20), however, Keleb et al.’s later series of papers provided the first comprehensive TSG studies, highlighting the TSE as a new alternative capable of directly substituting mixer granulators in the batch manufacturing of solid dosage forms (2). Typical flow-through of material in a TSE is given below (Fig. 3).

The initial step in the process of TSE after the material or a mixture of ingredients flows into the barrel is being conveyed to the mixing zone. The solids then undergo kneading with or without the addition of a liquid binder to form granules, and these agglomerates are then collected after being pumped out of the barrel. These granules may be milled to obtain the desired particle size fraction or can be used for direct compression unchanged (17).

Surpassing competition in the generic industry requires fewer deviations in production, higher yields, shortened time to market, and more profitable processes with lower operating, infrastructure, and capital costs. Majority of the generic pharmaceutical companies are trying to streamline their manufacturing process by implementing continuous manufacturing into their system so as to improve their existing products or upcoming generics. This review focuses on the details of a typical TSE—its design and components and applications in solid dosage form manufacturing with a highlight on the various forms of granulation that can be performed by TSE as a part of the continuous manufacturing process, as well as the influence of formulation and process on granule properties while using TSEs.

### Twin Screw Extruder Model Designs

A traditional twin screw extrusion setup consists of a drive unit, a heating thermocouple embedded extrusion barrel, a revolving shaft with screw elements, and a die. Indeed, a TSE delivers much more flexibility because the screw elements are easy to change to achieve the anticipated shear level and mixing speed. This variable shear experienced by the materials inside the extruder barrel may have varying effects, ranging from basic mixing of the API with the polymer to dispersing the substance into the polymer at a molecular level with the likelihood of drug-polymer interactions (21).

To some extent, the choice of an extruder for a given product depends on the stage of development of the product. Drugs and/or polymers might be scarcely available during the initial product development period. These times require small-scale extruders that have the potential to make do with small sample volumes (of the order of ~ 10–15 g), thus minimizing wastage. These small extruders are primarily used for basic formulation evaluation or raw material compatibility studies. HAAKE Minilab™ and HAAKE Minilab II Micro Compounder (Make—Thermo Scientific) and Omicron 10P (Make—Steer Engineering) are the leading small-scale twin screw extruders available commercially. Small bench space requirements, minimum dead space, and scalability make them highly versatile for the early formulation development process. The size of an extruder is determined by the screw diameter. Some of the varieties of TSEs have diameters of the range of 5 mm (Make—Three Tec), 10 mm (Make—Steer Engineering), 12 mm (Make—Brabender, Steer Engineering), 16 mm (Make—Leistritz, Thermo Scientific), 20 mm (Make—Steer Engineering), and even 40 mm (Make—Leistritz, Steer Engineering). Diameters of 5 mm to 12 mm are usually meant for lab-scale development work, 16 mm to 20 mm for pilot scale, and 20 mm to 40 mm are used for commercial-scale production. Some of the major continuous twin screw granulator manufacturers include GEA ConsiGma™ and Bohle BCG® Twin Screw Granulator integrated with a continuous granulator and drying line. The different extruders vary in their drive torque, barrel L/D ratio (20–60), screw elements design, barrel design (single or segmented), max achievable screw speed, price, the material used for construction, etc. and before buying a unit, the buyer should weigh every factor based on their requirement (22–25). Some of the TSEs quoted in research articles are mentioned in Table I.

**Table I:** List of TSEs Enlisted in Previous Reports

Manufacturer	Model	Screw diameter (mm)	References
Thermo Fisher	Prism EuroLab	16	(10, 26–30)
Thermo Fisher	Haake	16	(31)
Thermo Fisher	Pharma 24	24	(32)
APV Baker	MP 19 TC 25	19	(33)
GEA	ConsiGma 25	25	(34)
Leistritz	MIC 18/GG-40D	18	(35)
Leistritz	ZSE 27	27	(28, 36)
Leistritz	-	34 and 50	(37)
Steer Engineering Ltd	Omicron 10 P	10	(38)

### Individual Components of a Classic TSE

#### Drive Unit

The drive unit is one of the important components in the co-rotating TSE system. It is decisive for dependable operation. The drive unit includes an engine, safety clutch, and gearbox. The shear energy that helps to form agglomerates in the extruder is produced by the drive unit and transferred to the polymer mix by rotating screws. The mechanical power *P*_m_(W) that is introduced into the system is defined by the equation.:

$$P\mathrm{m }= \frac{2\pi n}{60} \times M$$

where *n* is the screw speed (rpm) and *M* is the torque (%).

The torque is a measure of the energy absorbed by a unit mass (specific energy) which is necessary to run a distinct process. The applied torque is therefore monitored as an important process parameter (39, 40).

#### Barrel and Feed Throat

The cylinder surrounding the extruder screws is the extruder barrel. It must endure substantially higher pressures and superior structural rigidity. Due to this, TSE barrels are generally made using a wear-resistant inner surface for their longevity (41). The barrel of the co-TSE is mounted as several short zones that are positioned one after the other to cover the screw length for its litheness. The overall length of the barrel is calculated in terms of L/D, where *L* is the screw length and *D* represents the outer diameter of a screw. The screws snugly fit within the top and bottom barrel, forming a restrained flow path for raw material, which is atypical of high shear granulator where the shear spread is less definitive. However, shear or wear and tear of the inner barrel linings depend on screw design and its precise alignment, without which solids might choke within the barrel (2).

The feed throat is a part of an extruder where the material is presented to the screw channel. It fits around the first few hops of the extruder screw. The barrel area around the feed throat is by and large water-cooled. This prevents polymer melting within the feed throat, causing flow congestion and conveying problems. The feed throat design should be such that material will flow into the extruder with bare minimum restriction (41).

For the screws to serve their purpose, the barrel design should be versatile enough to support user requirements, such as the inclusion of ports for solid material or external liquid feed and solvent vents to be positioned along the barrel corresponding to process requirements. Therefore, a segmented barrel is better acknowledged in the pharmaceutical industry (40).

#### Feeder

A continuous cascade of raw materials must be introduced into the extruder during a steady-state manufacturing run. This is accomplished by using feeders to meter solid, liquid, or gaseous materials through the process part (42). The feed rate typically sets the output rate and sustains formulation accuracy by controlling homogeneity (43, 44). Two major types of feeders are used along with an extruder: volumetric feeders and gravimetric feeders. Volumetric feeders are principally rpm-controlled and present a consistent material flow rate. These are less convenient if the bulk density of the raw material alters constantly. Gravimetric feeders (loss in weight) control the raw material flow rate based on the total weight of the material in the feeder. The throughput from the feeder is kept constant by a linear weight reduction over time. Extruders can be “starve-fed” or “flood-fed.” At steady-state, while being starve-fed, the input rate and the exit throughput are the same, and the accumulation within the barrel insides is insignificant. However, screw speed can play a major role in determining the raw material residence time distribution (41).

A continuous flow of fluids is achieved by pressuring the liquid input system to counteract obstruction due to solid materials within the barrel (43). The liquids directly enter the process segment utilizing closed liquid injection ports. These can be devised to work to operate at room or elevated temperatures, to feed solids with low glass transition temperature (*T*_g_) melted in the liquid state. Through proper equipment design, liquid feeding systems should be developed to pump fluids at sufficient pressures at a stable and reproducible rate (42).

Companies like Brabender Technologie, Coperion, Schenck AccuRate, and Geicke AG are among the leading manufacturers supplying gravimetric feeders fabricated to handle powders of varying densities and flow properties at varying dosage rates. Cartwright and coworkers described a crucial role of effective and accurate feeding of raw materials on granule manufacture by comparing across various scales as well as designs of commercially available gravimetric feeders (45, 46).

#### Screw and Screw Design

The TSE screw is the central core of the machinery. The screw rotation brings about a forward transfer, contributes significantly to polymer heating due to frictional shear, and elicits raw material homogenization (16). Being an exclusive process variable that affects granulation, screw design is conceivably the most dominant with regard to a TSG (2). The screws generally used in current TSGs consist of a shaft with individual screw components aligned in a predetermined sequence to provide an optimal screw configuration (40). The layout of screw elements in a screw design determines the method of granulation for a given TSG process. There are multiple screw element choices available at extruder vendors though granulation utilizes only a small subset of these (2).

The screws operating in the extruder are conventionally measured by using the L/D ratio (length of the screw divided by the outer screw diameter). Typical TSG screw lengths are in the range of 25–40 L/D (40).

**Fig. 4:**
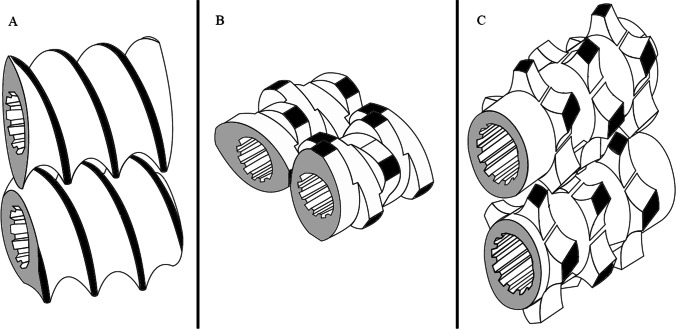
Common types of screw elements: **A** conveying element, **B** kneading element, **C** mixing element

The attributes related to each screw element based on its functions are discussed below. Screw parts can be graded loosely as forwarding, kneading, and mixing as shown in Fig. 4. It is not unusual for a screw element to have more than one of these characteristics, and maybe all three; however, a screw unit is usually primarily one of these simple types.

##### Forwarding/Conveying Elements

These units accomplish the task that the name implies and are used underneath barrel openings such as vents or feeders to push the material away from feed openings and transport it in a spiral, resembling Fig. 8, and pass it from one screw shaft to the other towards the first processing section where it may be compressed. The quantity of material being sent ahead is directly proportional to the screw speed and pitch angle of the actual screw element. Conveying by the elements generates the necessary push to introduce the materials into mixing zone elements. The classical types have the novelty self-wiping feature.

**Fig. 5:**
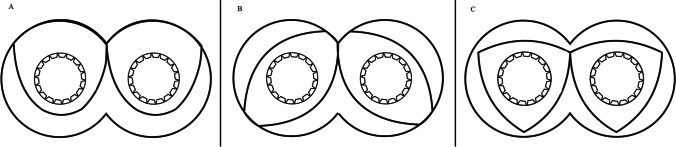
Conveying element profiles: **A** single lobed conveying elements, **B** bilobed conveying elements, **C** trilobed conveying elements

Conveying elements may be single lobed, bilobed, or trilobed, as seen in Fig. 5. Vendors supply these elements with varying pitches and lengths. They most often wipe the barrel with minimal clearances to avoid leaks over the screw tips. The greater the pitch angle, the higher the free volume in the screw. The pitch of the screw decides its loading efficiency. In the feed segment, a screw pitch of 1.5 to 2D is considered ideal for the raw material intake (47).

##### Kneading Elements (KE)

Kneading elements are chiefly used for the melting of polymers and efficient dispersion and mixing of fillers and binders used in a granulation run. KE are fabricated using multiple kneading discs that are offset at an explicit predetermined angle from each other. Conventional kneading elements are arranged symmetrically, much like the conveying elements, which means that the shape and location of the two sections on either of the screws are identical. Thus, the number of discs and their offset angle depend on each other.

**Fig. 6:**
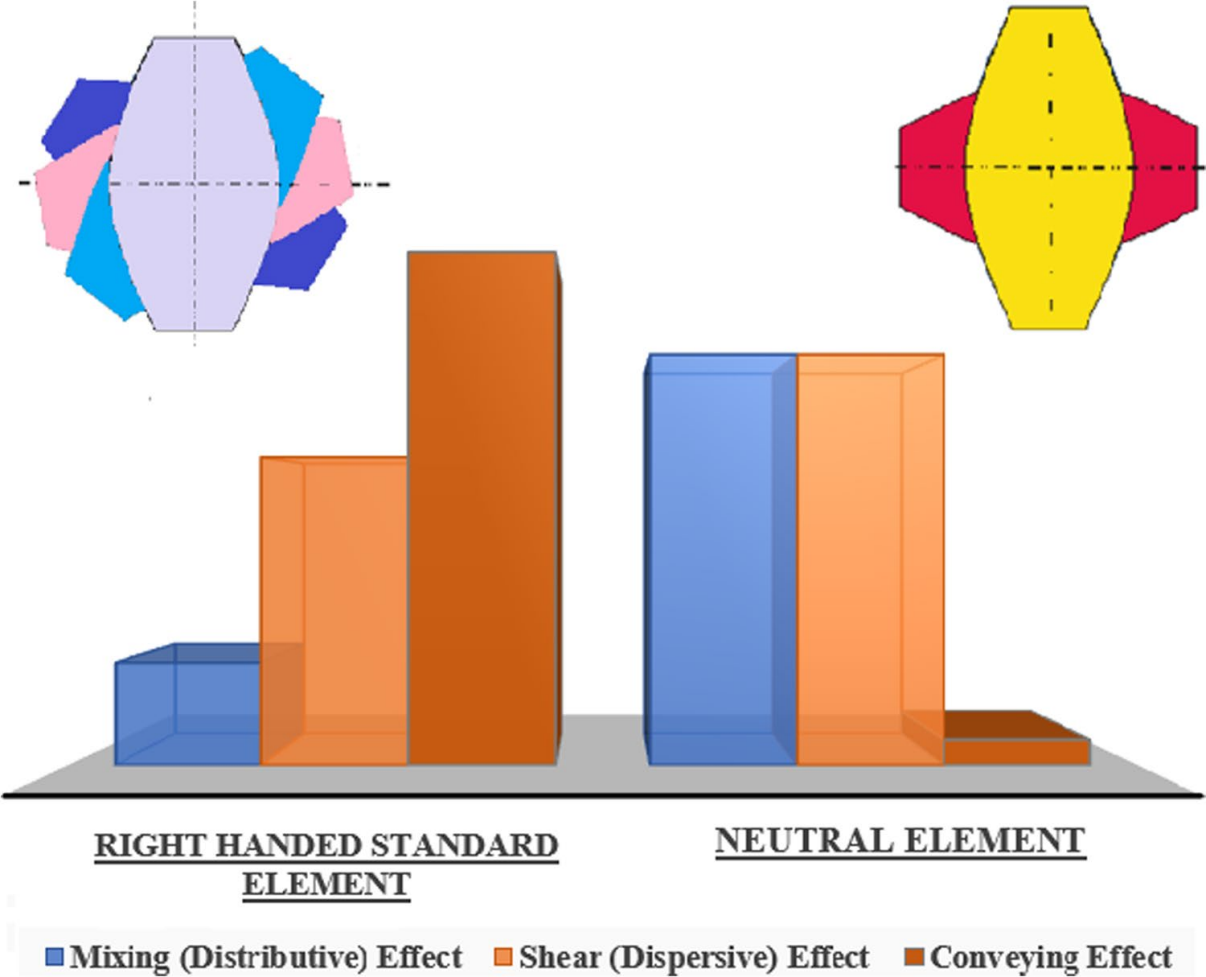
Comparison of mixing, shear, and conveying effect of kneading elements

The offset angle, number of discs, and width of the discs determine the extent of shear or conveying property that a kneading block would deliver. The larger the staggering angle among the kneading discs, the more open a kneading block is along its pivot that steps up its mixing effect. The downside to this is that the conveying capacity of the element is diminished. The kneading discs can be right-handed that convey material forward with minimal mixing, left-handed discs that create a melt plug and restrict material flow, and lastly neutral (i.e., 90°) that have no conveying characteristics but are quite effective mixers. Figure 6 describes the mixing, shear, and conveying efficacy of the right-handed and neutral kneading discs. The dispersive action of a kneading element is mainly because of the girth/width of the kneading disc. The greater the width of a kneading disc, the more are the chances of the material being forced between the gaps of the discs and the barrel wall, thus increasing the dispersive effect rather than distributive effect (12, 48).

##### Mixing Elements

Mixing elements can be functionally dispersive or distributive. Dispersive mixers are used to break down phase domains, droplets, and agglomerates and intimate mixing (44).

Combing mixing elements are the ones that perform both conveying and mixing functions simultaneously. Combing elements are akin to conveying elements but have lengthwise slits for distributive mixing and homogeneity enhancement. Comb mixing element is also mentioned in the text as distributive mixing elements (DME). DME relies on distributive mixing by cutting and recombining to allow interaction between the granular in-flow materials. Comparing its effects on granule properties, Li et al. documented that DME produces similar granules in the mixing or kneading area to those produced by kneading elements. The authors commented about no role of DME orientation on granule properties juxtaposing the reports by Sayin et al. proving superior granulation efficiency and thus superior granule size with a reverse configuration of DMEs where the orientation of DME is decided with respect to the incoming material feed that avoids the milling step (17, 26, 49, 50).

**Fig. 7:**
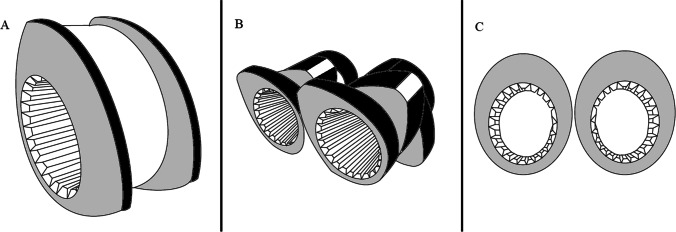
Special elements: **A** single-flighted kneading discs, **B** single-flighted kneading elements with narrow tip angles, **C** eccentric discs

The most widely used special elements today are for the premeditated application of elongational flows or specific shearing fields, or low shear plasticization. Some of the examples as shown in Fig. 7 include single-flighted kneading elements with narrow tip angles, eccentric discs, and single-flighted kneading discs (12).

##### Fractional Lobed Screw Elements

**Fig. 8:**
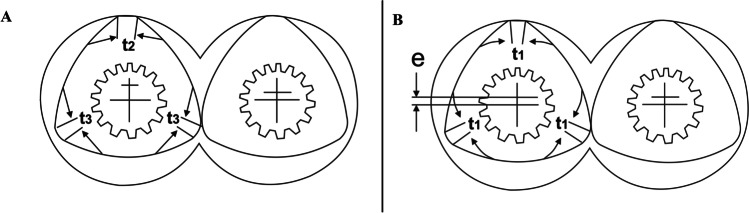
Trilobe design variations: **A** fractional trilobe design, **B** traditional trilobe design

Fractional lobed geometrical elements are an alternative style of screws for traditional elements in geometry. Based on the number of tips, the elements are classified as unilobed, bilobed, or trilobed (17) (represented by “T” in Fig. 8). The tip angle is a vital aspect of the twin screw extruders’ configuration. Awareness of the tip angle is extremely critical in recognizing the disparity in geometry between the unique fractional lobe elements (Fig. 8A) and traditional elements (Fig. 8B). With the increased tip angle, an element reveals an almost circular shape, thus allowing a constant free space to be retained between the element and the screw barrel, which traditional components cannot achieve (17, 51).

Some of the types of fractional lobe elements include fractional kneading block (FKB), a right-handed fractional kneading block (RFKB), eccentric fractional kneading block (EKB), fractional mixing element (FME), eccentric fractional mixing element (EME), continuous mixing element (CME), 3Lobe right-hand screw element (3RSE), 3Lobe dynamic stir element (3DSA), and melt formation element (MFE) (52).

### Material of Construction (MOC)

This is one area in which TSEs vary from conventional equipment used in pharmaceutical processes. The “interaction” between raw material and equipment metal must be considered while selecting the screw and barrel MOC. MOC of machinery for pharmaceutical uses must be extremely durable and inert. This is why high-quality stainless steel is typically used that is resistant to corrosion. The most widely used corrosion-resistant material is 304 or 316 stainless steel. However, it is relatively fragile and can easily be weakened. Also, high mechanical stress is often involved in screws rotating at fairly high speed (up to 1200 rpm in some cases) with minimum clearances between the screw and the barrel lining (typically 0.2 mm). Therefore, 304 or 316 stainless steel cannot be used for the manufacture of screw elements or barrel housing. To avoid these disadvantages, they are usually made of sturdy grades of stainless alloys similar to those used in tablet punches manufacturing (e.g., stainless steel type 440B), which are not as resistant to corrosion but are stronger and last longer. The elements used in the pharmaceutical industry do not typically have a surface coating such as nickel or chrome plating as there is a possibility of coating to sliver off contaminating the product. Other components such as feeders and hardware can be made of 304 or 316 stainless steel. Other product contact surfaces shaft seals or barrel die seals should be made up of materials similar to Teflon that are inert in nature (44, 53, 54).

### Coupling Process Analytical Technology (PAT) with TSE

The objective of PAT is to ensure finished product quality by timely in-line measurements of CPP and CQAs via process understanding and better manufacturing process control. The basis on which PAT guideline was introduced by the USFDA was to encourage and support innovation and utilization of risk-based approaches by the pharmaceutical industry, regulatory, and quality agencies. Unlike the traditional consideration of precise process time, PAT model claims that an end point has been reached when the specified material characteristics have been attained; however, this does not imply that the process time is not considered. During production, a process window of acceptable process times should be reviewed, and strategies for resolving large deviations from accepted process times should be established. Control methods that validate the performance of the manufacturing process are one of the approaches to comply with cGMP standards. Monitoring process parameters like feed rate, speed of screw shaft, and barrel temperature and the capability to combine PAT that enables CQA management in-line during the process is the crucial aspect of continuous manufacturing. These regulatory criteria will further push the pharmaceutical industry to move away from batch production and towards continuous manufacturing. The integration of PAT tools and model-based process knowledge will be the future of quality control and process understanding. With the use of multivariate data analysis techniques in conjunction with process sensors to measure physical and chemical data in real time, PAT technologies may be developed to increase process knowledge. Application of PAT during pharmaceutical development can potentially enhance product quality and safety and reduce batch variations and losses (15, 55–59).

Moisture content, API content uniformity, blend uniformity, granule size distribution, and solid state of the active ingredient are the major CQAs that need to be monitored in a twin screw granulation process using PAT measurement tools. Some of the most commonly used nondestructive, noninvasive PAT tools include ultraviolet/visible spectroscopy, NIR and MIR spectroscopy, Raman spectroscopy, focused beam reflectance measurement (FBRM), and special filtering velocimetry (SFV), among others (60–62).

Harting and Kleinebudde assessed Raman spectroscopy, an in-line PAT measurement tool for continuous API estimation during twin screw wet granulation. The duo developed and used statistical models to predict the API concentration in the applied range, which exhibited optimum results. The developed models were also used to quantify API during the split feeding process successfully, which verified the superior mixing efficiency of the twin screw granulator/extruder. These experiments determined the application of Raman spectroscopy to simplify continuous manufacturing run by efficient in-line drug substance content uniformity assessment (61, 63).

Pauli et al. developed three methods using NIR spectroscopy to predict the D10, D50, and D90 PSD fractions of dried granules in a continuous twin screw wet granulation process. This approach was used to monitor abrupt changes in granules PSD in real time along with other parameters like moisture content of the granules and API content. Using internal as well as external validation datasets, they were able to demonstrate the robustness of the method with the data showing a maximum variation of 174 µm in *D*_90_ range of 748–2298 µm and minimum variation of 17 µm in *D*_10_ range of 20–234 µm. The robustness of methods against common LOD and API content variations has also been demonstrated by a decent correlation between actual and predicted values. NIR proved that due to the rapid response time, they could improve process control by effective monitoring of PSD, LOD, and API content in in-line and off-line conditions of continuous manufacturing (64).

Kumar et al. mainly investigated the use of focused beam reflectance measurement (FBRM) as an in-line, real-time tool to evaluate the particle size distribution and develop a relationship between FBRM data and PSD data from sieve analysis during a continuous granulation drying and milling process. Superimposing FBRM data and sieve analysis showed a similar bimodal graph for all the experimental runs that is statistically significant. This similarity helps in developing a feedback mechanism in a continuous process to adjust CPPs in case of any deviations. Changes in screw speed or mill speed did not show any impact on granule size or micromeritic properties suggesting a wide operating range (65).

Schlindwein et al. demonstrated the ability to use UV–visible spectroscopy as an in-line, real-time PAT tool for hot melt extrusion. Quality by Design (QbD) was applied to observe the effects of die temp, screw rpm, feed rate, and API (piroxicam) concentration on the absorbance of the hot melt extrudes. Real-time color lightness and visible region absorbance were used as CQAs to identify solubility limits. UV–visible spectroscopy was found to be easy to interpret with rapid delivery of results with high sensitivity. Based on the results obtained, a possible efficient design space was developed with acceptable processing conditions. The concentration of drug moiety and temperature were found to be chief critical parameters. In-line UV–visible spectroscopy was found to be useful in HME as the solubility of the drug in the excipient matrix can be visually quantified. However, this technique can only be used for active moieties that have a distinct footprint in the ultraviolet–visible region (66).

## Applications of Twin Screw Extruder

### Granulation Technology

Granulation, a popular practice in the pharmaceutical industry, is considered one of the most important unit operations in solid dosage form development. It is carried out prior to the tableting to improve the overall powder properties. Small fine or coarse particles undergo size enlargement during the granulation process. An ideal granulation will contain all the constituents of the mix in the correct proportion in each granule, and segregation of granules will not occur. Thus, the ultimate aim of granulation is to increase powder mixture flow and compressibility. The granulation process can also:

- Ensure uniform distribution of the API.
- Reduce particle size distribution in the granulated blend.
- Improve the bulk density of the powder blend to minimize fine generation.
- Improve the solubility and dissolution attributes of the finished product.

Wet granulation commonly involves a liquid binder that is sprayed (fluid bed granulation) or drained slowly (high shear granulation) over a disturbed bed of powder where individual particles layer over each other on the wetting layer, in a process dubbed as agglomeration (10, 67–69). It is a critical technology that allows ill-flowing, poorly compressible, or poorly dissolving active substances to be transformed into elegant and efficient finished products such as tablets and capsules (70). Granulation technique can be effortlessly converted into a continuous phase using extrusion technology that involves two screws revolving within a barrel to constantly convey, blend, and agglomerate wet particles, where the energy required for material densification is provided by shear and/or heating. On the basis of the density of the material that exits from the extruder can either be used immediately (e.g., extrusion process run without die) or necessitates an additional size reduction stage to comminute the dense extrudates to granules within a particle size range (18, 44, 71). A comparison between traditional batch granulation techniques (high shear granulation and fluid bed granulation) and twin screw granulation is given in Table II. Twin screw granulation can be branched into different groups depending on the use of the binders and type of granulating solvents used in the process, such as twin screw steam granulation, twin screw foam granulation, twin screw wet granulation, and twin screw melt granulation. A summary of various twin screw granulation processes is given in Table III.

**Table II:** A Comparison Between Traditional Batch Granulation Techniques (High Shear Granulation and Fluid Bed Granulation) and Twin Screw Granulation

Parameters	High shear granulation	Fluid bed granulation	Twin screw granulation
Granule shape	Dense and almost spherical	Dense and spherical in nature	Irregularly shaped with uniform pores
Granule porosity	Reduced porosity resulting in compact granules	Reduced surface area with less porous granule structure	Higher surface area and porous granules resulting in better dissolution characteristics
Granulation efficacy	Can result in localized over-granulation due to binder solvent addition	Can result in over-granulation due to irregular spray pattern	Better granulation as contact of solvent with excipients is minimum due to reduced residence time
Granule consistency	Gross inconsistency of wet granules due to impeller shear	Granule to granule inconsistency may be seen	Consistent granule quality
Process time	Long process time	Longer process time	Reduced process time
Scalability	Difficult to scale up	Difficult to scale up	Can be scaled up on the same machinery by increasing the process time

**Table III:** Summary of Different Types of Twin Screw Granulation Process

TSFG	TSSG	TSMG	TSWG
Uses externally generated foam as the binder	Uses externally generated steam as the granulating agent	Molten binder acts as granulating agent	Uses water or organic solvent as the granulation solvent
Improved spreadability and enhances surface area per unit volume of binder solution sprayed	Higher diffusion rate of granulating liquid as compared to other techniques	Solvent free—no drying step	Reduced use of granulating solvent as compared to batch granulation
Better granule size distribution with high porosity	Better synchronization of granulation process initiation	Reduced fines as compared to other procedures	
Ideal for water-sensitive and thermo-sensitive APIs due to reduced exposure to heat and solvent			
Shorter granulation time as compared to traditional granulation			

#### Twin Screw Foam Granulation (TSFG)

TSFG is a new technology that is evolved to diminish excess wetting in the wet granulation method during the initial addition of a binder to the dry raw material mix. Tan et al. investigated the approach of foam granulation (FG) on a stationary powder bed and found that the nucleating efficiency was better compared to the drop addition method because of its lower soak-to-spread ratio. The process is wet granulation, but the binder liquid is foamed right before it is added to the dry raw material mix. Here, a binding solution might contain a foaming agent that may be dissolved, or other excipients, such as cellulose derivatives, can serve as both binder and a foaming agent (72, 73). High shear granulation experiments have assessed the FG method finding benefits linked to the reduced requirement of binder solution quantity, a lower process run time, and better consistency of binder inclusion without injector choking (74–77). FG by TSE is capable of delivering a uniform granular product of excipients and API with high consistency in a relatively short timeframe. TSFG requires reduced binder concentration to manufacture a granule with similar properties compared to the conventional wet granulation process needing less energy input during drying and limited downstream milling.

Thompson et al. contrasted direct fluid binder injection in a TSE with the inclusion of foam in wet granulation (foam granulation) to observe its effects on finished granule properties. This study aimed at investigating the importance of foamed binder solution as an improvement in binder delivery system for continuous wet granulation in a TSE and examining the actions of granulation at high production rates during which the filled volume in barrel and screws was high. The chances of reduced binder use during the trial run were related to the foam binder’s low soak-to-spread ratio, which uniformly wetted the powder without over-saturation. The end product of the process produced by FG was comparable in terms of granulate strength to traditional wet granulation (78).

Li et al. compared four different API formulations with different hydrophobicities to determine their effects on twin screw foam-based granulation. Based on the results, the author described the hydrophobic API to be caged by the hydrophilic binders and other ingredients, thus restricting its spread state as it was in the initial wetting stage throughout the process and had limited impact (79).

Weatherley et al. devised research comparing FG and WG (wet granulation) in a continuous TSE to review the impact of the binder chemistry and concentration on the granulation. In this experiment, various critical quality attributes were explored, including the concentration of the binders in the aqueous binder solutions, the forms of cellulose derivatives used (varying both chemistry and molecular weight), and the binder addition process. Overall, the result revealed that TSFG in a TSE was similar to the conventional granulation technique in that most of the variables studied did not negatively or favorably influence the distribution of particle size or granule flow properties. Foam granulation allowed the spreading properties of foams with a higher molecular weight binder creating coarser granules compared to lower molecular weights. The results also show that foam granulation helped minimize Carr’s compressibility index by approximately 24% with all samples showing excellent to fair flow properties according to USP chapter < 1174 > (73, 80).

#### Twin Screw Melt Granulation (TSMG)

Melt granulation (MG) uses a molten binder for aggregation of raw material particles instead of a granulated liquid, thus avoiding the drying phase, reducing process time and energy consumption (81). MG based on TSE has gained acceptance in the pharmaceutical world over the last decade, based on its ability to achieve improved process control and more homogenous product quality. Since thermal energy is generated in TSMG from the barrel heaters and by friction between particles and the screw or barrel surface, it is far more effective in contrast to batch manufacturing. TSMG’s distinctive heating system facilitates uniform heat dispersion and minimizes drug degradation in case it is thermolabile (82). Also, the limited area within the extruder barrel allows effective mass and heat transfer, resulting in good product consistency (45).

TSMG involves the raw material input blend containing API, binder, and other excipients being conveyed through the screw shaft, where the barrel temperature is maintained near the Tg of the binder to ensure its melting and uniform distribution between the particles, leading to agglomeration. Generally, a binder with low Tg is preferred for TSMG which can be used at low concentrations. Binder selection plays a vital role in TSMG. A binder with a Tg or melting point lower than other ingredients is considered better in TSMG. However, effective TSMG heating also allows the use of thermoplastic polymers with high melting points as a thermal binder (61–63, 83).

MG gained its popularity due to the availability of a wide variety of excipients that were approved by the FDA. Some of the major excipients or carrier systems include polyvinylpyrrolidone and its copolymers, vinyl acetate copolymers, polyethylene glycols (PEG), cellulose ethers, polyethylene oxide, and waxes. The basic prerequisite for the use in melt granulation is the thermoplastic nature of the polymers or that of the respective formulations (84–90).

Lakshman et al. used TSMG to improve the compressibility of metformin hydrochloride. The study compares TSMG to other granulation methods like wet and dry granulation. In this study, the author melt granulated the high-dose drug—metformin HCl (MP: 224 °C)—with a cellulosic binder having low Tg (Tg: 130 °C). It was done in such a way that the overall processing temperature of the TSE is not greater than the degradation temperature of API. Various finished product specifications such as tablet hardness, friability, weight variation, and dissolution were explored and compared. The TSMG process was found to be superior to conventional granulation. Batch-to-batch variability did not occur with consistent granulation being seen even at low moisture content. TSMG obtained better compaction at lower moisture as opposed to using a solvent. The hardness of the tablet was measured between 150 and 400 N, with direct reference to friability. Thus, the author used TSE to maintain stable and efficient processes while making high dosage formulations (91).

Patil et al. investigated the application of TSMG to prepare sustained release formulation of a drug with pH-dependent solubility. They aimed to create a pH-independent drug release formulation using TSG process using stearic acid as the release retardant binder in combination with hydrophobic and hydrophilic polymers. Parameters such as micromeritics of the granules, particle size distribution, tablet properties evaluation, and their release kinetics were analyzed and compared for their similarity and dissimilarity. The findings showed that the granules which were prepared using stearic acid as the binder demonstrated good flow and compressibility with minimal fines. The combination of hydrophobic and hydrophilic polymers with stearic acid in a matrix formulation exhibited sustained release of the drug with almost 90% release in 24 h. This research demonstrated TSE’s capacity to fabricate readily compressible melt granules continuously (92).

Keen et al. established a continuous TSMG method for the manufacture of tramadol HCl controlled release granules using glyceryl behenate as the lipid excipient. The granulation process developed did not completely melt the lipid owing to short residence time. Apart from validating TSMG as a viable process for producing ready to compress granules, the author explained the effect of the barrel temperature profile, throughput rate, and initial particle size on the final granule particle size distribution. Continuous twin screw melt granulation ensued a tablet formulation with a robust dissolution profile and stability (93).

#### Twin Screw Steam Granulation (TSSG)

Steam granulation (SG) is a modern method of wet granulation, requiring the use of steam instead of conventional water as granulation solvent. Steam granulation aims to solve the challenges of the handling of granulation fluids by using steam as the granulating agent instead of water. The high steam diffusion rate allows it to mix with the raw materials intimately, while the steam condensation on the particle surface tends to create a film of binder fluid right at the particle agglomeration and granule nucleation interface. Compared to conventional wet granulation, the use of steam in the wet granulation will greatly minimize the volume of water required and thereby the overall work period. The consistent spread of steam on particles can help to build granules not only with greater uniformity, but also with higher porosity, which will lead to enhanced dissolution profile (94–97).

Steam-aided granulation was described by Cavallari et al. where the research aim was to report the findings of its effect on *in vitro* dissolution rate and its comparison with water-aided wet granulation. Based on the authors’ findings, it was clear that steam addition resulted in increased porosity of the granules due to high thermal energy of steam and the experimental conditions where steam granulation higher fractal dimension and a more irregular surface. They attributed the higher rate of dissolution in steam granulation to the higher porosity and greater surface area in the steam granulate content relative to wet granulation (98).

In contrast to Cavallari et al.’s conventional granulation system, Ghike et al. filed a patent for a twin screw processor with a steam feeder that would inject steam as the granulation initiator to form granules. The method was designed to obtain dry granules directly from the twin screw processor without any additional drying stage by adding an optimal quantity of steam relative to raw material feed, sufficient to granulate the input material but not exceeding it. Agglomeration and uniform distribution of steam occur simultaneously within the granulation zone of the twin screw processor. The zone is also responsible for granule sizing, which eliminates the need for a separate milling phase. Using 2.5–3% steam, the author granulated paracetamol with 2.5% binder, disintegrant, and 20% diluent. The granulated material’s final moisture content was found to be 2.52%, with uniform particle size granules. The author describes a versatile process to manufacture granules with ideal properties and high granulation yields continuously. These operations are carried out at low barrel temperatures and thereby reduce the amount of moisture in the final granules; hence, the manufacturing process may be optimized for the granulation of moisture- and/or heat-sensitive substances (99).

#### Twin Screw Wet Granulation (TSWG)

Among other methods, twin screw wet granulation (TSWG) is the most appropriate imminent continuous unit operation for the pharmaceutical manufacturing industry efficient enough to produce granules using reduced granulating fluid quantity as compared to traditional mixer granulators with the added advantage of consistency, better control over its stability, minimum residence time, controlled output, and more adaptable to in-line analytical tools. TSWG could substitute the current techniques for batch granulation, making the granulation process more versatile, reproducible, and cost-effective. In this twin screw granulation technique, raw material input enters the equipment where the blend is conveyed through the barrel. Granulation of raw material blend takes place because of granulating solvent like water or binder solution. Use of specific screw elements like kneading blocks and distributive elements allows agglomeration as well as the milling of the granules along the length of the barrel. Use of minimum quantity of solvent means wet granules can be dried within the barrel length using barrel temperature and thus additional drying step is circumvented. The various factors that affect the final granule quality that acts as critical quality attributes for the TSWG process include feed rate, liquid to solid (L/S) ratio, barrel fill volumes, processing temperatures, and screw elements (17, 27, 31, 35, 50, 100–103).

Dhenge et al. (2011) investigated the effect of varying feed rates from 2 to 6 kg/h on the residence time and properties of TSWG granules while maintaining other variables such as the liquid to solid ratio (0.3), screw design (two kneading zones), screw speed (400 rpm), and barrel temperature (25 °C). A nonfunctional API (sodium chloride) was combined with a solid binder (hydroxypropyl cellulose) at a concentration of 5%. The granulating agent was water. Feed rates were found to be inversely proportional to residence time distributions, owing primarily to barrel fill stage. However, the findings indicated that increasing the feed rate resulted in a greater concentration of fines of good strength. This is primarily due to material cramming caused by high feed rates and short residence times, which results in increased attrition between the wet mass and the screws and barrel. Thus, this article emphasizes the importance of feed rate on granule efficiency (104).

Ito and Kleinebudde investigated the effect of granulation process temperature on particle size distribution (PSD) during TSWG. The impact of granulation temperature on PSD varies depending on the binder chemistry, binder addition method, diluents, and L/S ratio. At higher temperatures, the granulation solvent or solution decreased in viscosity leading to better distribution within the barrel and helped in homogenous granulation and thus narrow PSD. Keeping L/S ratio constant, the PSD graph was broad at lower barrel temperatures. PSD of granules was bimodal when water soluble diluent was used as a result of higher effective L/S ratio leading to larger lumps. Use of di-calcium phosphate resulted in narrow PSD range. The authors also described the use of binder solution where its viscosity led to inefficient spreading within the powder bed leading to bimodal distribution of granules. Hence, the granulation temperature is an important parameter for obtaining mono-modal PSD in TSG from these results (31).

Portier et al. explored the effects of screw configurations (kneading elements thickness and fraction of KE elements), barrel fill levels (screw speed), and L/S ratios on model formulations using microcrystalline cellulose (MCC) and lactose monohydrate (LM) as diluents, HPMC as the dry binder, and metformin hydrochloride and mebendazole as model APIs. According to the results obtained, for formulations containing only LM as a filler, greater L/S ratio factor effects were found, expressed by the larger fines fraction similar to research data by Hwang et al. Owing to the lower specific feed load and thus barrel fill rate, higher screw speeds resulted in significantly smaller granules. Use of KE of smaller thickness (1/6 L/D) results in better attrition and increased fines. This is because the complete cutting plane is separated into more individual elements, thus increasing shear action. However, this resulted in poor flow of granules as compared to KE of higher thickness (1/4 L/D). To test TSWG’s maximum potential, the author recommended more studies on poorly soluble high-capacity APIs and different kinds of diluents (103, 105).

Saleh et al. studied the relationship of binder delivery on granule properties and its effect on granule properties using conveying and kneading elements. The author investigated dry binder delivery, binder solution delivery, and a 50:50 mixture of dry binder and binder solution. When a dry binder was used in conjunction with only conveying elements, the resulting granules had a small size distribution with low oversize and fines, in contrast to when a liquid binder solution was used, which is attributed to the granulating solvent’s low viscosity and effective dispersion within the powder bed. Additionally, this resulted in an increase in residence time and torque. Due to the limited number of conveying elements used, reduced shear resulted in poor agglomeration. Granule size increased by using a liquid binder with kneading elements due to increased shearing during granulation, thus ensuring a more intimate interaction between the powder mix and the granulation fluid. On the opposite, uniform liquid delivery with kneading elements results in minimal interaction between granules and effective attrition, resulting in a reduction in oversized granules (106).

Granule attributes were characterized by Sayin et al. varying distributive mixing elements (DME) directionally and spatially in TSWG. Keeping all other variables constant, the DME direction has a greater influence on the PSD of granules than its spatial orientation. A contrast between forward and reverse configurations has found that reverse orientation yields better granule qualities due to more efficient mixing and binder exchange. In comparison to kneading elements, DME facilitates the liquid distribution and PSD of granules without affecting their structural integrity or density. Granules of reverse-placed DME are stable enough to eliminate the need for downstream processing by efficient drying and better control over PSD. They also have superior micromeritic properties (47).

Apart from the abovementioned reports, many have studied in detail about various aspects affecting granules in a twin screw wet granulation process such as effect of a specific screw element on granule properties and solvent distribution (27, 107), impact of temperature on wettability of HPMC in TSWG (28), importance of barrel fill level on critical attributes of granules (29, 102), impact of varying viscosity and surface tension of binder liquid on granules (30), achieving content uniformity in ultra-low dose (108), effect of binder chemistry on granule size distribution (109), effect of varying excipient characteristics (diluents and binders) on TSE (110), and visualization of flow and mixing patterns in a TSE (111).

### Other Applications

#### Three-Dimensional Printing

The usual perception of “one-size-fits-all” dosage form for every individual is difficult to achieve due to high unevenness in genetics, ethnicity, gender, age, and patient weight (112). Three-dimensional (3D) printing of drug formulations allows the pharmacists and doctors a chance to personalize the dose, bearing in mind patient-to-patient variability and possibly improving safety and efficacy for some medications which traditional dosage form manufacturing process can hardly do (113–115). 3D printing is a powder, liquid, and/or extrusion-based additive manufacturing technique that utilizes 5 major methods: (i) selective laser sintering (SLS), (ii) stereolithography (SLA), (iii) semisolid extrusion printing, (iv) thermal inkjet printing, and (v) 3D extrusion printing such as fused deposition modeling (FDM) (116). FDM uses a thermoplastic polymer filament, usually formed by twin screw melt extrusion (TSME), as starting material. The filament is pushed into the printer’s heated block by a geared tool to soften or melt. In this way, subsequent material layers can be mounted on the 3D printing plate through a nozzle. The overlaid layers fuse and embed to each other after cooling, enabling one to get the final 3D product (117). Many 3D printing methods were designed to generate many dosage formulations, have the advantages of being adjustable and cost efficient, requires limited distance, computer-controlled and flexible drug delivery. Some of the various drug delivery systems manufactured using 3D printing include controlled/sustained release tablets/scaffolds, implantable systems, intragastric floating tablets, multicompartment capsules, orodispersible films, bilayer tablets, liquid capsules, and wound dressings (118). Table IV gives a list of manuscripts that mention various techniques of 3D printing used to develop various dosage forms.

**Table IV:** Various Reported Techniques of 3D Printing

Technique	Dosage form	API	References
Selective laser sintering	Square film and tablet	Amlodipine, lisinopril dihydrate	(119)
	Miniprintlets (mini tablets)	Paracetamol, ibuprofen	(120)
Stereolithography	Ring/cylinder-shaped printlets (tablets)	Paracetamol, naproxen, caffeine, aspirin, prednisolone, and chloramphenicol	(121)
	Ring/cylinder-shaped printlets (tablets)	Irbesartan, atenolol, hydrochlorothiazide, and amlodipine	(122)
	Scaffolds (3D bioprinting)	Simvastatin	
Semisolid Extrusion printing	Tablet	Aspirin, hydrochlorothiazide, pravastatin, atenolol, and ramipril	(123)
Thermal inkjet printing	Orodispersible films	Triiodothyronine (T3) and thyroxine (T4)	(124)
Fused deposition model (FDM)	Polypill tablets	Indapamide, rosuvastatin calcium, amlodipine besylate, and lisinopril dihydrate	(125)
	Multilayered tablet, DuoCaplet	Paracetamol, caffeine	(126)
	Capsular device using injection molding	NA	(127)
	Immediate release tablets	Carvedilol, haloperidol	(128)
	Controlled release tablets	Budesonide	(126)
	Implantable systems	Indomethacin	(129)
	Sustained release scaffolds	Carbamazepine	(130)
	Orodispersible films	Aripiprazole	(131)
	Liquid capsules	Theophylline, dipyridamole	(132)
	Multicompartment capsules	Caffeine	(133)
	Bilayer tablets	Metformin hydrochloride and Glimepiride	(134)
Fused deposition model (FDM)	Wound dressings	Zinc, copper, silver	(135)
	Intragastric floating tablets	Domperidone	(136)
Low temperature fused deposition model (FDM)	Immediate release tablets	Ramipril	(137)
Direct powder extrusion	Abuse deterrent controlled release tablets	Tramadol	(138)
Digital light processing	Printlets (tablets)	Ibuprofen	(139)
Stencil printing	Orodispersible discs	Haloperidol	(140)
Embedded 3D printing	Chewable dosage form	Paracetamol and ibuprofen	(141)
Pressure-assisted microsyringe (PAM)	Controlled release bilayer tablets	Guaifenesin	(142)

#### Applications in Nutraceuticals

TSEs also have been used on a very wide scale in food, nutraceutical, and polymer industries apart from pharmaceutical industries. TSE has been majorly used in the nutraceutical industry for encapsulation of flavonoids, flavors, volatile oils, heat-sensitive biologically active compounds, readily oxidizable components, etc.

Yilmaz et al. researched on encapsulation of a hydrophobic bioactive compound in a carbohydrate matrix. The authors used glycerol as a plasticizer where increasing amounts of the plasticizer resulted in a finer more uniform dispersion of the lipophilic compound within the hydrophilic matrix. Process parameters such as screw speed, throughput, barrel temperature, and screw configuration had an imperative role to play in the development and encapsulation of dispersed phase. Thus, the research establishes the role of formulation and extrusion process parameters in modifying the discrete hydrophobic phase in a hydrophilic core (143).

Chang et al. used a twin screw extruder (HAAKE PolyLab system) for preparing a stable dispersion of ascorbic acid (AA) in a matrix of maltodextrin using water as a plasticizer. The objective here was to prepare AA extrudates that can deliver its nutritional properties without being affected by other ingredients or processing conditions. AA being readily oxidizable, the water content of the finished extrudates played a major role in the development. Scanning electron microscopy and X-ray diffraction data indicated complete miscibility of the core in the matrix suggesting a molecular level dispersion (144).

Bernard H Van Lengerich filed an international patent where the invention describes a continuous process using TSEs for fabricating distinct control release particles that encapsulate a biologically active compound without destroying their activity. The author, in two separate experiments, successfully encapsulated the microorganism *Lactobacillus acidophilus* and enzyme Phytase, into nongelatinized wheat starch where the blend was mixed with water in the twin screw extruder forced through the die at low temperature and low shear. The extrudate was then cut into pellets that remain shelf stable with the encapsulated component biologically active. The matrix in discussion is nongelatinized wheat starch which is used to control the release rate of the encapsulant by delaying its dissolution. Also, this being a molecular level interaction, the author also expects higher bioavailability (145).

Khor et al. succeeded in encapsulating quercetin using the technique of hot melt extrusion so as to taste mask its bitterness. Carnauba wax, shellac, and zein were blended individually with 70% polyphenolic flavonoid and extruded through TSE (Prism EuroLab 16—Thermo Scientific), in these taste masking trials. *In vitro* bitterness assessment using a taste sensing system confirmed its masked taste. Similar *in vitro* digestion profiles were seen in carnauba wax and shellac-coated quercetin particles deeming it suitable for further development. The research proved TSE of its efficiency in taste masking plant-based polyphenols (146).

#### Twin Screw Extrusion and Nanotechnology

In drug delivery research, nanonization of insoluble BCS class 2 and 4 drugs is a highly investigated formulation approach. Polymeric nanoparticles, solid lipid nanoparticles, inorganic nanoparticles, nanosuspensions, and nanocrystals have already been reported for controlled release drug delivery by increasing bioavailability by modification of dissolution rate and/or tissue distribution in the oral drug delivery field. These are primarily obtained by the use of advanced size reduction techniques (147, 148). Applying twin screw extrusion technology for developing nano-sized delivery platforms is an exciting venture in combination with high pressure homogenization and sonication to produce solid lipid nanoparticles continuously.

Baumgartner et al. use TSE (MICRO 27 GL Leistritz GmbH) as a novel manufacturing technique to continuously convert stable nanosuspensions into a solid oral formulation. Using soluplus® as the melt matrix, a stable nanosuspension of phenytoin, prepared using a surfactant as a stabilizer, was mixed with the molten polymer and water from the suspension was immediately degassed using vacuum to obtain solid extrudates which were cut into pellets. Characterization techniques such as transmission electron microscopy and atomic force microscopy revealed that the phenytoin nanocrystals were uniformly dispersed in the polymer matrix with no change in crystallinity of the drug substance. This study aptly demonstrates tailored TSE’s capability to produce a complex solid dosage form continuously (148).

Gajera et al. published an article where TSE (Omicron 10P—Steer Engineering) was used to dry an amorphous nanosuspension. Clotrimazole nanosuspension was prepared by antisolvent technique with soluplus® solution as the aqueous phase. The nanosuspension was then fed at a constant rate into the extruder which was maintained at a certain temperature (110–130 °C). The output consisted of the drug embedded in the soluplus® matrix. Quality by design application led to an optimized drying process that helped in achieving higher yields and minimum moisture content and optimum redispersibility in the final product (147).

Koo et al. developed iron sulfate nanoparticles using TSE. The nanoparticles were formulated using surfactants such as Span 80, Tween 80, and polyethylene glycol 6000. The process involved mixing all the ingredients and feeding into the TSE with a die maintained at a temperature above the Tg of PEG 6000. The extrudates were then pulverized for further use. The fabricated nanoparticle dispersion was in the size range of 350–400 nm with a narrow size distribution and spherical shape. The cell line studies displayed improved efficacy of the colloidal nanoparticles compared to iron sulfate. The article appropriately describes the use of TSE to develop targeted drug delivery techniques by oral route (149).

Lee et al. succeeded in developing zinc supplement formulations containing zinc sulfate nanocomposites. The formulation was developed by blending zinc sulfate and soluplus and continuously feeding it to the TSE. The extruder was maintained at a temperature of 100–110 °C with a die and the extrudates were milled before further use. Characterization of the nanocomposites suggests that ZnSO4 was homogenously dispersed throughout the inert polymer with a mean particle size of 75 nm and low polydispersity. The authors established a stable formulation using twin screw extrusion technology without significant toxicity, which indicates that the formulation developed can be used *in vivo* effectively and safely (150).

Overcoming the limitations of methods combining TSE with homogenization, sonication, etc. (151, 152), Guo et al. developed a one-step continuous extrusion method for the production of highly concentrated lipid nanocarrier dispersions (~ 60% lipids). At an elevated temperature, a crude dispersion of lipids and surfactants is prepared in an aqueous phase. After heating the crude dispersion, it was fed into a modified TSE equipped with a cooling chamber at the end. Within the first half of the extruder, the coarse dispersion is heated, mixed, and homogenized, and then rapidly cooled in the second half to collect the extrudate below room temperature. The authors achieved a size range of less than 100 nm by increasing the lipid concentration from 20 to 50%. The article discusses the potential for twin screw extrusion technology to assist in the development of nanoparticle dispersions without requiring subsequent size reduction measures (153).

Badge et al. developed topical gel formulation of ibuprofen dispersed in solid lipid nanoparticles using twin screw extrusion. The author applied design of experiments approach to prepare the formulation. Stable carbopol gel formulation with 0.48% drug-loaded solid lipid nanoparticles with a size of about 60 nm showed better drug release, drug disposition, and edema control as compared to ibuprofen gel. The study explains the potential of twin screw extrusion technique in the construction of lipid nanoparticle dispersions incorporated in topical gels (154).

## Challenges Associated with Twin Screw Extrusion

Some of the challenges that would need addressing in the field of twin screw extrusion are the following:

- Exposure to localized temperature and stress may lead to undesirable polymorphic changes of the active moiety.
- Extremely temperature sensitive drugs can be chemically degraded leading to loss of potency.
- Feasible add-ons may be required for downstream processing of granules (i.e., milling sieving).
- Pharmaceutical industry is prudent about regulatory filings while introducing novel technologies due to difficulty in getting approval from various regulatory bodies.

## Other Reported Continuous Unit Operations

### Continuous Feeding

Consistently feeding powder is one of the key criteria for continuous manufacturing of any designed product. To generate quality products, continuous powder processing requires a steady input stream of raw ingredients. This means that the initial feeding stage is important to the entire production process. Blackshields and Crean provided a brief overview about the principle and mechanism of continuous feeding with some good case studies. Continuous tablet production lines can be as simple as continuous dry powder mixing and continuous direct compression. This is where continuous powder feeders are vital to the overall operation of the continuous tabletting line. Even if the mixer and tablet press work well, the tablet quality will change if the in-flow composition is highly variable. Thus, proper control of each bulk material’s feed rate is critical (155, 156).

### Continuous Blending

Ervasti et al. investigated continuous mixing and direct compression for prolonged release tablet manufacturing. The authors adjusted the mixing speed and the duration of processing to achieve a final product with robust properties (assay, weight, tensile strength). The authors found that it is critical to strike a balance between raw material characteristics and process parameters in order to generate high-quality products consistently (156).

Colon and colleagues established the validity of a near-infrared spectroscopic technique for the continuous blending process. The precision and accuracy of the NIR technique for predicting the end point of the blending process were examined by the authors. An established model was utilized to monitor a continuous blending process for 3 min, during which samples were analyzed using NIR and UV spectroscopy. It was discovered that the standard deviation ranged from 0.47 to 0.53%, indicating that NIR was a suitable technique for investigating the variability of blends at various phases of production (157).

### Continuous Finished Product Manufacturing

Pauli et al. devised a continuous twin screw wet granulation method in which pre-lubricated blends were fed into a twin screw granulator through gravimetric feeders to produce dry powder. The wet granules were dried in a fluid bed dryer and then transported to milling equipment for size reduction before being compressed into tablets through an automated powder transfer system. The API content was determined using NIR spectroscopy throughout feeding, end of granulation, drying, and tableting. Frequent step testing throughout the production process, as well as the determination and maintenance of a standard residence time for each operation, would eliminate any deviation from given specifications (64).

The FDA assists new technologies of continuous manufacturing that assists in pharmaceutical modernization and to bring impending benefits both to the industry and to patients. Over the past decade, significant research has proven the upper hand of continuous manufacturing over batch processes by offering flexibility in scale-up, qualitative, and monetary gains such as condensing machine trails by assimilating several processes and lowering capital costs, pliable to real-time quality testing by the use of PAT technologies (158).

## Conclusion

This review focuses on the topic of twin screw extruder and how the equipment has spearheaded the concept of continuous manufacturing. Variations in individual screw units or formulation attributes can modify the functionality of hot melt extrusion to WG, MG, FG, and SG. Agglomerates obtained using TSWG are the preferred alternatives to the long-established batch wet granulation process. The ability of TSWG process to function and deliver the granules at ambient temperatures without extra drying stage works as an added advantage which can be exploited for thermolabile drugs. TSWG, TSMG, TSFG, and TSSG can be implemented for sustained release formulations of low- or high-dose medications, stability, and solubility enhancement. In conclusion, state-of-the-art twin screw granulation process holds imminent promise as the best bet in the field of granulation in the pharmaceutical world. In future, there are several areas that can be explored in the field of continuous manufacturing using TSG by coupling QbD approach with the novelty of mathematical computational modeling to attenuate the complexities in granulation technology. The plastic and food industry embraced and utilized twin screw granulation decades ago, and it is the pharmaceutical sector’s turn to incorporate it into continuous manufacturing process. This proven processing technique would replace batch granulation processing through technical expertise and experience in the field. The progress of continuous twin screw granulation would continue as long as there is a coordinated effort and commitment by the pharmaceutical industry, equipment manufacturers, excipients suppliers, and regulatory bodies to advance continuous manufacturing.

## References

[1] Meng W, Dvořák J, Kumar R, Hofmeister R, Štěpánek F, Ramachandran R, Muzzio FJ (2019). Continuous high-shear granulation: mechanistic understanding of the influence of process parameters on critical quality attributes via elucidating the internal physical and chemical microstructure. Adv Powder Technol.

[2] Thompson MR (2015). Twin screw granulation – review of current progress. Drug Dev Ind Pharm.

[3] Galbraith SC, Cha B, Huang Z, Park S, Liu H, Meyer RF, Flamm MH, Hurley S, Zhang-Plasket F, Yoon S (2019). Integrated modeling of a continuous direct compression tablet manufacturing process: a production scale case study. Powder Technol.

[4] Tian G, Koolivand A, Arden NS, Lee S, O’Connor TF (2019). Quality risk assessment and mitigation of pharmaceutical continuous manufacturing using flowsheet modeling approach. Comput Chem Eng.

[5] Badman C, Trout BL (2015). Achieving continuous manufacturing May 20–21 2014 continuous manufacturing symposium. J. Pharm. Sci..

[6] Lee SL, O’Connor TF, Yang X, Cruz CN, Chatterjee S, Madurawe RD, Moore CMV, Yu LX, Woodcock J (2015). Modernizing pharmaceutical manufacturing: from batch to continuous production. J Pharm Innov.

[7] Badman C, Cooney CL, Florence A, Konstantinov K, Krumme M, Mascia S, Nasr M, Trout BL (2019). Why We need continuous pharmaceutical manufacturing and how to make it happen. J Pharm Sci.

[8] Vanhoorne V, Vervaet C (2020). Recent progress in continuous manufacturing of oral solid dosage forms. Int J Pharm.

[9] Van Snick B, Holman J, Vanhoorne V, Kumar A, De Beer T, Remon JP, Vervaet C (2017). Development of a continuous direct compression platform for low-dose drug products. Int J Pharm.

[10] Dhenge RM, Fyles RS, Cartwright JJ, Doughty DG, Hounslow MJ, Salman AD (2010). Twin screw wet granulation: granule properties. Chem Eng J.

[11] Manne ASN, Hegde AR, Raut SY, Rao RR, Kulkarni VI, Mutalik S (2021). Hot liquid extrusion assisted drug-cyclodextrin complexation: a novel continuous manufacturing method for solubility and bioavailability enhancement of drugs, Drug Deliv. Transl Res.

[12] Kohlgrüber K, Bierdel M (2008). Co-rotating twin-screw extruder: fundamentals, technology, and applications.

[13] M.M. Crowley, F. Zhang, M.A. Repka, S. Thumma, S.B. Upadhye, S. Kumar Battu, J.W. McGinity, C. Martin, Pharmaceutical applications of hot-melt extrusion: part I, Drug Dev. Ind. Pharm. 33 (2007) 909–926. 10.1080/03639040701498759 17891577

[14] Sarabu S, Bandari S, Kallakunta VR, Tiwari R, Patil H, Repka MA (2019). An update on the contribution of hot-melt extrusion technology to novel drug delivery in the twenty-first century: part II. Expert Opin Drug Deliv.

[15] M. Maniruzzaman, ed., Practical guide to hot-melt extrusion: continuous manufacturing and scale-up, Smithers Rapra Technology Ltd, Shawbury, Shrewsbury, Shropshire, 2015.

[16] Rauwendaal CJ (1981). Analysis and experimental evaluation of twin screw extruders. Polym Eng Sci.

[17] Bandari S, Nyavanandi D, Kallakunta VR, Janga KY, Sarabu S, Butreddy A, Repka MA (2020). Continuous twin screw granulation – an advanced alternative granulation technology for use in the pharmaceutical industry. Int J Pharm.

[18] Vervaet C, Remon JP (2005). Continuous granulation in the pharmaceutical industry. Chem Eng Sci.

[19] Lindberg N-O, Tufvesson C, Holm P, Olbjer L (1988). Extrusion of an effervescent granulation with a twin screw extruder, Baker Perkins MPF 50 D. Influence on Intragranular Porosity and Liquid Saturation. Drug Dev Ind Pharm.

[20] Gamlen MJ, Eardley C (1986). Continuous extrusion using a Raker Perkins MP50 (multipurpose) extruder. Drug Dev Ind Pharm.

[21] Thiry J, Krier F, Ratwatte S, Thomassin J-M, Jerome C, Evrard B (2017). Hot-melt extrusion as a continuous manufacturing process to form ternary cyclodextrin inclusion complexes. Eur J Pharm Sci.

[22] A. Loxley, Devices and implant systems by hot-melt extrusion, in: D. Douroumis (Ed.), Hot-Melt Extrus. Pharm. Appl., Wiley, Hoboken, NJ, n.d.: p. 301.

[23] Bohle | Twin-Screw Granulation | Pharmaceutical Industry, LB Bohle Maschinen Verfahr. GmbH. (2020). https://lbbohle.com/machines-processes/granulation/twin-screw-granulation-bcg/ (accessed September 25, 2020).

[24] Lab-extruders, (n.d.). http://www.steerworld.com/lab-extruders.html (accessed September 25, 2020).

[25] ConsiGma® Granulation and Compression (GC) Lines, GEA Eng. Better World. (n.d.). http://www.gea.com/en/products/tablet-presses/continuous-tableting-lines/consigma-continuous-tablet-line.jsp (accessed September 25, 2020).

[26] Li J, Pradhan SU, Wassgren CR (2019). Granule transformation in a twin screw granulator: effects of conveying, kneading, and distributive mixing elements. Powder Technol.

[27] El Hagrasy AS, Litster JD (2013). Granulation rate processes in the kneading elements of a twin screw granulator. AIChE J.

[28] Liu Y, Thompson MR, O’Donnell KP, Grasman NS (2016). Effect of temperature on the wetting behavior of hydroxypropyl methylcellulose in a twin-screw granulator. Powder Technol.

[29] Lute S, Dhenge R, Salman A (2018). Twin Screw Granulation: An Investigation of the Effect of Barrel Fill Level. Pharmaceutics.

[30] Dhenge RM, Cartwright JJ, Hounslow MJ, Salman AD (2012). Twin screw wet granulation: Effects of properties of granulation liquid. Powder Technol.

[31] Ito A, Kleinebudde P (2019). Influence of granulation temperature on particle size distribution of granules in twin-screw granulation (TSG). Pharm Dev Technol.

[32] Osorio JG, Sayin R, Kalbag AV, Litster JD, Martinez-Marcos L, Lamprou DA, Halbert GW (2017). Scaling of continuous twin screw wet granulation. AIChE J.

[33] Van Melkebeke B, Vervaet C, Remon JP (2008). Validation of a continuous granulation process using a twin-screw extruder. Int J Pharm.

[34] J. Vercruysse, D. Córdoba Díaz, E. Peeters, M. Fonteyne, U. Delaet, I. Van Assche, T. De Beer, J.P. Remon, C. Vervaet, Continuous twin screw granulation: Influence of process variables on granule and tablet quality, Eur. J. Pharm. Biopharm. 82 (2012) 205–211. 10.1016/j.ejpb.2012.05.010. 10.1016/j.ejpb.2012.05.010 22687571

[35] Liu H, Ricart B, Stanton C, Smith-Goettler B, Verdi L, O’Connor T, Lee S, Yoon S (2019). Design space determination and process optimization in at-scale continuous twin screw wet granulation. Comput Chem Eng.

[36] Thompson MR, Sun J (2010). Wet Granulation in a Twin-Screw Extruder: Implications of Screw Design. J Pharm Sci.

[37] Shah U (2005). Use of a modified twin-screw extruder to develop a high-strength tablet dosage form. Pharm Technol.

[38] Ajjarapu S, Rangappa S, Shankar VK, Shettar A, Kumar HNS, Kulkarni VI, Repka MA, Murthy SN (2020). A Rapid Tool to Optimize Process Variables for Continuous Manufacturing of Metronidazole Ointment Using Melt Extrusion Technique. AAPS PharmSciTech.

[39] G.R. Huber, Twin-screw extruders, Extruders Food Appl. (2000) 81–114.

[40] Douroumis D (2012). Hot-melt extrusion: pharmaceutical applications.

[41] Rauwendaal C, Gramann PJ (2014). Polymer extrusion.

[42] A. Haser, J.C. DiNunzio, C. Martin, J.W. McGinity, F. Zhang, Melt Extrusion, in: R.O. Williams III, A.B. Watts, D.A. Miller (Eds.), Formul. Poorly Water Soluble Drugs, Springer International Publishing, Cham, 2016: pp. 383–435. 10.1007/978-3-319-42609-9_9.

[43] S. Shah, M.A. Repka, Melt Extrusion in Drug Delivery: Three Decades of Progress, in: M.A. Repka, N. Langley, J. DiNunzio (Eds.), Melt Extrus., Springer New York, New York, NY, 2013: pp. 3–46. 10.1007/978-1-4614-8432-5_1.

[44] Ghebre-Sellassie I, Martin C, Zhang F, DiNunzio J (2018). Pharmaceutical extrusion technology.

[45] M. Lodaya, M. Thompson, Continuous oral solid dose manufacture, in: Pharm. Extrus. Technol., Second, Taylor & Francis Group, New York, NY, n.d.: pp. 337–361.

[46] Cartwright JJ, Robertson J, D’Haene D, Burke MD, Hennenkamp JR (2013). Twin screw wet granulation: Loss in weight feeding of a poorly flowing active pharmaceutical ingredient. Powder Technol.

[47] Wi. Thiele, Twin screw extrusion and screw design, in: Pharm. Extrus. Technol., Second, CRC Press, Boca Raton, FL, 2003: pp. 71–93.

[48] Teixeira C, Gaspar-Cunha A, Covas JA (2012). Flow and heat transfer along the length of a co-rotating twin screw extruder. Polym-Plast Technol Eng.

[49] Djuric D, Kleinebudde P (2008). Impact of screw elements on continuous granulation with a twin-screw extruder. J Pharm Sci.

[50] Sayin R, El Hagrasy AS, Litster JD (2015). Distributive mixing elements: towards improved granule attributes from a twin screw granulation process. Chem Eng Sci.

[51] B. Padmanabhan, CO-ROTATING TWIN-SCREW ELEMENTS, (n.d.) 10.

[52] I. Bhushan, B. Padmanabhan, V. Rao, V. Kulkarni, C. Chincholi, R. Ghike, R. Ganeshan, Processor and a process for granulation of powders, (2019).

[53] J. Perdikoulias, T. Dobbie, Die Design, in: I. Ghebre-Sellassie, C. Martin, F. Zhang, J.C. DiNunzio (Eds.), Pharm. Extrus. Technol., Second, CRC Press, Boca Raton, FL, n.d.: p. 430.

[54] A. Dreiblatt, Installation, Commissioning, and Qualification, in: I. Ghebre-Sellassie, C. Martin, F. Zhang, J.C. DiNunzio (Eds.), Pharm. Extrus. Technol., Second, CRC Press, Boca Raton, FL, n.d.: p. 430.

[55] Guidance for Industry PAT - a framework for innovative pharmaceutical development, manufacturing, and quality assurance, (n.d.) 19.

[56] Andrews GP, Jones DS, Senta-Loys Z, Almajaan A, Li S, Chevallier O, Elliot C, Healy AM, Kelleher JF, Madi AM, Gilvary GC, Tian Y (2019). The development of an inline Raman spectroscopic analysis method as a quality control tool for hot melt extruded ramipril fixed-dose combination products. Int J Pharm.

[57] R.V. Barenji, Y. Akdag, B. Yet, L. Oner, Cyber-physical-based PAT (CPbPAT) framework for Pharma 4.0, Int. J. Pharm. 567 (2019) 118445. 10.1016/j.ijpharm.2019.06.036. 10.1016/j.ijpharm.2019.06.036 31226474

[58] R.W. Bondi, J.K. Drennen, Quality by Design and the Importance of PAT in QbD, in: Sep. Sci. Technol., Elsevier, 2011: pp. 195–224. 10.1016/B978-0-12-375680-0.00005-X.

[59] Lang B, McGinity JW, Williams RO (2014). Hot-melt extrusion – basic principles and pharmaceutical applications. Drug Dev Ind Pharm.

[60] Hitzer P, Bäuerle T, Drieschner T, Ostertag E, Paulsen K, van Lishaut H, Lorenz G, Rebner K (2017). Process analytical techniques for hot-melt extrusion and their application to amorphous solid dispersions. Anal Bioanal Chem.

[61] Harting J, Kleinebudde P (2019). Optimisation of an in-line Raman spectroscopic method for continuous API quantification during twin-screw wet granulation and its application for process characterisation. Eur J Pharm Biopharm.

[62] Zhong L, Gao L, Li L, Zang H (2020). Trends-process analytical technology in solid oral dosage manufacturing. Eur J Pharm Biopharm.

[63] Harting J, Kleinebudde P (2018). Development of an in-line Raman spectroscopic method for continuous API quantification during twin-screw wet granulation. Eur J Pharm Biopharm.

[64] Pauli V, Roggo Y, Kleinebudde P, Krumme M (2019). Real-time monitoring of particle size distribution in a continuous granulation and drying process by near infrared spectroscopy. Eur J Pharm Biopharm.

[65] Kumar V, Taylor MK, Mehrotra A, Stagner WC (2013). Real-time particle size analysis using focused beam reflectance measurement as a process analytical technology tool for a continuous granulation–drying–milling process. AAPS PharmSciTech.

[66] Schlindwein W, Bezerra M, Almeida J, Berghaus A, Owen M, Muirhead G (2018). In-Line UV-Vis Spectroscopy as a Fast-Working Process Analytical Technology (PAT) during Early Phase Product Development Using Hot Melt Extrusion (HME). Pharmaceutics.

[67] R. Gokhale, Y. Sun, A. Shukla, High-Shear Granulation, in: Handb. Pharm. Granulation Technol., Second, Taylor & Francis Group, New York, NY, n.d.: pp. 191–228.

[68] Thapa P, Tripathi J, Jeong SH (2019). Recent trends and future perspective of pharmaceutical wet granulation for better process understanding and product development. Powder Technol.

[69] S. Shanmugam, Granulation techniques and technologies: recent progresses, BioImpacts BI. 5 (2015) 55–63. 10.15171/bi.2015.04. 10.15171/bi.2015.04 PMC4401168 25901297

[70] T. Dürig, K. Karan, Binders in Wet Granulation, in: Handb. Pharm. Wet Granulation, Elsevier, 2019: pp. 317–349. 10.1016/B978-0-12-810460-6.00010-5.

[71] Keleb EI, Vermeire A, Vervaet C, Remon JP (2002). Continuous twin screw extrusion for the wet granulation of lactose. Int J Pharm.

[72] Tan MXL, Wong LS, Lum KH, Hapgood KP (2009). Foam and drop penetration kinetics into loosely packed powder beds. Chem Eng Sci.

[73] Weatherley S, Thompson MR, Sheskey PJ (2013). A study of foam granulation and wet granulation in a twin screw extruder. Can J Chem Eng.

[74] Keary CM, Sheskey PJ (2004). Preliminary Report of the Discovery of a New Pharmaceutical Granulation Process Using Foamed Aqueous Binders. Drug Dev Ind Pharm.

[75] P. Shesky, C. Keary, D. Clark, Scale-up trials of foam-granulation technology: high shear., Pharm. Technol. 31 (n.d.) 94–108.

[76] S.L. Cantor, S. Kothari, O.M.Y. Koo, Evaluation of the physical and mechanical properties of high drug load formulations: Wet granulation vs. novel foam granulation, Powder Technol. 195 (2009) 15–24. 10.1016/j.powtec.2009.05.003.

[77] Tan MXL, Hapgood KP (2011). Foam granulation: Binder dispersion and nucleation in mixer-granulators. Chem Eng Res Des.

[78] Thompson MR, Weatherley S, Pukadyil RN, Sheskey PJ (2012). Foam granulation: new developments in pharmaceutical solid oral dosage forms using twin screw extrusion machinery. Drug Dev Ind Pharm.

[79] Li H, Thompson MR, O’Donnell KP (2015). Examining drug hydrophobicity in continuous wet granulation within a twin screw extruder. Int J Pharm.

[80] P. Sheskey, C. Keary, U. Shrestha, J. Becker, Use of a novel foam granulation technique to incorporate low drug loading into immediate-release tablet formulations, in: Annu. Meet. Expo. Am. Assoc. Pharm. Sci., 2004: pp. 7–9.

[81] Monteyne T, Vancoillie J, Remon J-P, Vervaet C, De Beer T (2016). Continuous melt granulation: Influence of process and formulation parameters upon granule and tablet properties. Eur J Pharm Biopharm.

[82] Kittikunakorn N, Liu T, Zhang F (2020). Twin-screw melt granulation: Current progress and challenges. Int J Pharm.

[83] Van Melkebeke B, Vermeulen B, Vervaet C, Remon JP (2006). Melt granulation using a twin-screw extruder: A case study. Int J Pharm.

[84] Vasanthavada M, Wang Y, Haefele T, Lakshman JP, Mone M, Tong W, Joshi YM, Serajuddin ATM (2011). Application of Melt Granulation Technology Using Twin-screw Extruder in Development of High-dose Modified-Release Tablet Formulation. J Pharm Sci.

[85] Breitenbach J (2002). Melt extrusion: from process to drug delivery technology. Eur J Pharm Biopharm.

[86] Tantishaiyakul V, Kaewnopparat N, Ingkatawornwong S (1999). Properties of solid dispersions of piroxicam in polyvinylpyrrolidone. Int J Pharm.

[87] G. Zingone, M. MONEGHINI, P. Rupena, D. Vojnovic, Characterization and dissolution study of solid dispersions of theophylline and indomethacin with PVP/VA copolymers, STP Pharma Sci. 2 (1992) 186–192.

[88] Perissutti B, Newton JM, Podczeck F, Rubessa F (2002). Preparation of extruded carbamazepine and PEG 4000 as a potential rapid release dosage form. Eur J Pharm Biopharm.

[89] Zhang F, McGinity JW (1999). Properties of Sustained-Release Tablets Prepared by Hot-Melt Extrusion. Pharm Dev Technol.

[90] Monteyne T, Adriaensens P, Brouckaert D, Remon J-P, Vervaet C, De Beer T (2016). Stearic acid and high molecular weight PEO as matrix for the highly water soluble metoprolol tartrate in continuous twin-screw melt granulation. Int J Pharm.

[91] Lakshman JP, Kowalski J, Vasanthavada M, Tong W-Q, Joshi YM, Serajuddin ATM (2011). Application of Melt Granulation Technology to Enhance Tabletting Properties of Poorly Compactible High-Dose Drugs. J Pharm Sci.

[92] Patil H, Tiwari RV, Upadhye SB, Vladyka RS, Repka MA (2015). Formulation and development of pH-independent/dependent sustained release matrix tablets of ondansetron HCl by a continuous twin-screw melt granulation process. Int J Pharm.

[93] Keen JM, Foley CJ, Hughey JR, Bennett RC, Jannin V, Rosiaux Y, Marchaud D, McGinity JW (2015). Continuous twin screw melt granulation of glyceryl behenate: Development of controlled release tramadol hydrochloride tablets for improved safety. Int J Pharm.

[94] L. Rodriguez, C. Cavallari, N. Passerini, B. Albertini, M.L. González-Rodríguez, A. Fini, Preparation and characterization by morphological analysis of diclofenac/PEG 4000 granules obtained using three different techniques, Int. J. Pharm. 242 (2002) 285–289. 10.1016/S0378-5173(02)00189-8. 10.1016/s0378-5173(02)00189-8 12176265

[95] A.S. Narang, S.I.F. Badawy, Emerging Paradigms in Pharmaceutical Wet Granulation, in: Handb. Pharm. Wet Granulation, Elsevier, 2019: pp. 825–840. 10.1016/B978-0-12-810460-6.00025-7.

[96] E. Jannat, A.A. Arif, M. Hasan, A. Bin, H.A. Rashid, Granulation techniques & its updated modules, (n.d.) 8.

[97] Suresh P, Sreedhar I, Vaidhiswaran R, Venugopal A (2017). A comprehensive review on process and engineering aspects of pharmaceutical wet granulation. Chem Eng J.

[98] C. Cavallari, B. Abertini, M.L. González-Rodríguez, L. Rodriguez, Improved dissolution behaviour of steam-granulated piroxicam, Eur. J. Pharm. Biopharm. 54 (2002) 65–73. 10.1016/S0939-6411(02)00021-8. 10.1016/s0939-6411(02)00021-8 12084504

[99] R. Ghike, V. Kulkarni, I. Bhushan, H. Sen, B. Padmanabhan, V. Rao, A PROCESS AND APPARATUS FOR CONTINUOUS GRANULATION OF POWDER MATERIAL, US 2018 / 0214835, n.d.

[100] Shirazian S, Zeglinski J, Darwish S, Kuhs M, Albadarin AB, Croker DM, Walker GM (2018). Continuous twin screw wet granulation: The combined effect of process parameters on residence time, particle size, and granule morphology. J Drug Deliv Sci Technol.

[101] Seem TC, Rowson NA, Ingram A, Huang Z, Yu S, de Matas M, Gabbott I, Reynolds GK (2015). Twin screw granulation — A literature review. Powder Technol.

[102] Meier R, Moll K-P, Krumme M, Kleinebudde P (2017). Impact of fill-level in twin-screw granulation on critical quality attributes of granules and tablets. Eur J Pharm Biopharm.

[103] Portier C, Pandelaere K, Delaet U, Vigh T, Di Pretoro G, De Beer T, Vervaet C, Vanhoorne V (2020). Continuous twin screw granulation: A complex interplay between formulation properties, process settings and screw design. Int J Pharm.

[104] Dhenge RM, Cartwright JJ, Doughty DG, Hounslow MJ, Salman AD (2011). Twin screw wet granulation: Effect of powder feed rate. Adv Powder Technol.

[105] Hwang K-M, Cho C-H, Yoo S-D, Cha K-I, Park E-S (2019). Continuous twin screw granulation: Impact of the starting material properties and various process parameters. Powder Technol.

[106] Saleh MF, Dhenge RM, Cartwright JJ, Hounslow MJ, Salman AD (2015). Twin screw wet granulation: Binder delivery. Int J Pharm.

[107] Lute SV, Dhenge RM, Hounslow MJ, Salman AD (2016). Twin screw granulation: Understanding the mechanism of granule formation along the barrel length. Chem Eng Res Des.

[108] Démuth B, Fülöp G, Kovács M, Madarász L, Ficzere M, Köte Á, Szabó B, Nagy B, Balogh A, Csorba K, Kaszás G, Nagy T, Bódis A, Marosi G, Nagy ZK (2020). Continuous Manufacturing of Homogeneous Ultralow-Dose Granules by Twin-Screw Wet Granulation, Period. Polytech. Chem Eng.

[109] Vanhoorne V, Janssens L, Vercruysse J, De Beer T, Remon JP, Vervaet C (2016). Continuous twin screw granulation of controlled release formulations with various HPMC grades. Int J Pharm.

[110] Willecke N, Szepes A, Wunderlich M, Remon JP, Vervaet C, De Beer T (2017). Identifying overarching excipient properties towards an in-depth understanding of process and product performance for continuous twin-screw wet granulation. Int J Pharm.

[111] Lee KT, Ingram A, Rowson NA (2012). Twin screw wet granulation: The study of a continuous twin screw granulator using Positron Emission Particle Tracking (PEPT) technique. Eur J Pharm Biopharm.

[112] Algahtani MS, Mohammed AA, Ahmad J, Saleh E (2020). Development of a 3D Printed Coating Shell to Control the Drug Release of Encapsulated Immediate-Release Tablets. Polymers.

[113] Cohen JS (1999). Ways to minimize adverse drug reactions: Individualized doses and common sense are key. Postgrad Med.

[114] Fanous M, Gold S, Hirsch S, Ogorka J, Imanidis G (2020). Development of immediate release (IR) 3D-printed oral dosage forms with focus on industrial relevance. Eur J Pharm Sci.

[115] Mathew E, Pitzanti G, Larrañeta E, Lamprou DA (2020). 3D Printing of Pharmaceuticals and Drug Delivery Devices. Pharmaceutics.

[116] Keikhosravi N, Mirdamadian SZ, Varshosaz J, Taheri A (2020). Preparation and characterization of polypills containing aspirin and simvastatin using 3D printing technology for the prevention of cardiovascular diseases. Drug Dev Ind Pharm.

[117] Melocchi A, Uboldi M, Cerea M, Foppoli A, Maroni A, Moutaharrik S, Palugan L, Zema L, Gazzaniga A (2020). A Graphical Review on the Escalation of Fused Deposition Modeling (FDM) 3D Printing in the Pharmaceutical Field. J Pharm Sci.

[118] Gültekin HE, Tort S, Acartürk F (2019). An Effective Technology for the Development of Immediate Release Solid Dosage Forms Containing Low-Dose Drug: Fused Deposition Modeling 3D Printing. Pharm Res.

[119] S.J. Trenfield, H.X. Tan, A. Goyanes, D. Wilsdon, M. Rowland, S. Gaisford, A.W. Basit, Non-destructive dose verification of two drugs within 3D printed polyprintlets, Int. J. Pharm. 577 (2020) 119066. 10.1016/j.ijpharm.2020.119066 31982555

[120] Awad A, Fina F, Trenfield SJ, Patel P, Goyanes A, Gaisford S, Basit AW (2019). 3D printed pellets (miniprintlets): A novel, multi-drug, controlled release platform technology. Pharmaceutics.

[121] Robles-Martinez P, Xu X, Trenfield SJ, Awad A, Goyanes A, Telford R, Basit AW, Gaisford S (2019). 3D printing of a multi-layered polypill containing six drugs using a novel stereolithographic method. Pharmaceutics.

[122] X. Xu, P. Robles-Martinez, C.M. Madla, F. Joubert, A. Goyanes, A.W. Basit, S. Gaisford, Stereolithography (SLA) 3D printing of an antihypertensive polyprintlet: Case study of an unexpected photopolymer-drug reaction, Addit. Manuf. 33 (2020) 101071.

[123] Khaled SA, Burley JC, Alexander MR, Yang J, Roberts CJ (2015). 3D printing of five-in-one dose combination polypill with defined immediate and sustained release profiles. J Controlled Release.

[124] Alomari M, Vuddanda PR, Trenfield SJ, Dodoo CC, Velaga S, Basit AW, Gaisford S (2018). Printing T3 and T4 oral drug combinations as a novel strategy for hypothyroidism. Int J Pharm.

[125] Pereira BC, Isreb A, Forbes RT, Dores F, Habashy R, Petit J-B, Alhnan MA, Oga EF (2019). ‘Temporary Plasticiser’: A novel solution to fabricate 3D printed patient-centred cardiovascular ‘Polypill’architectures. Eur J Pharm Biopharm.

[126] Goyanes A, Wang J, Buanz A, Martínez-Pacheco R, Telford R, Gaisford S, Basit AW (2015). 3D printing of medicines: engineering novel oral devices with unique design and drug release characteristics. Mol Pharm.

[127] Maroni A, Melocchi A, Parietti F, Foppoli A, Zema L, Gazzaniga A (2017). 3D printed multi-compartment capsular devices for two-pulse oral drug delivery. J Controlled Release.

[128] C. Wei, N.G. Solanki, J.M. Vasoya, A.V. Shah, A.T. Serajuddin, Development of 3D Printed Tablets by Fused Deposition Modeling Using Polyvinyl Alcohol as Polymeric Matrix for Rapid Drug Release, J. Pharm. Sci. (2020). 10.1016/j.xphs.2020.01.015 32004538

[129] Holländer J, Genina N, Jukarainen H, Khajeheian M, Rosling A, Mäkilä E, Sandler N (2016). Three-dimensional printed PCL-based implantable prototypes of medical devices for controlled drug delivery. J Pharm Sci.

[130] Lim SH, Chia SMY, Kang L, Yap KY-L (2016). Three-dimensional printing of carbamazepine sustained-release scaffold. J Pharm Sci.

[131] W. Jamróz, M. Kurek, E. \Lyszczarz, J. Szafraniec, J. Knapik-Kowalczuk, K. Syrek, M. Paluch, R. Jachowicz, 3D printed orodispersible films with Aripiprazole, Int. J. Pharm. 533 (2017) 413–420. 10.1016/j.ijpharm.2017.05.052 28552800

[132] Okwuosa TC, Soares C, Gollwitzer V, Habashy R, Timmins P, Alhnan MA (2018). On demand manufacturing of patient-specific liquid capsules via co-ordinated 3D printing and liquid dispensing. Eur J Pharm Sci.

[133] Melocchi A, Parietti F, Maccagnan S, Ortenzi MA, Antenucci S, Briatico-Vangosa F, Maroni A, Gazzaniga A, Zema L (2018). Industrial development of a 3D-printed nutraceutical delivery platform in the form of a multicompartment HPC capsule. AAPS PharmSciTech.

[134] Gioumouxouzis CI, Baklavaridis A, Katsamenis OL, Markopoulou CK, Bouropoulos N, Tzetzis D, Fatouros DG (2018). A 3D printed bilayer oral solid dosage form combining metformin for prolonged and glimepiride for immediate drug delivery. Eur J Pharm Sci.

[135] Muwaffak Z, Goyanes A, Clark V, Basit AW, Hilton ST, Gaisford S (2017). Patient-specific 3D scanned and 3D printed antimicrobial polycaprolactone wound dressings. Int J Pharm.

[136] Chai X, Chai H, Wang X, Yang J, Li J, Zhao Y, Cai W, Tao T, Xiang X (2017). Fused Deposition Modeling (FDM) 3D Printed Tablets for Intragastric Floating Delivery of Domperidone. Sci Rep.

[137] Kollamaram G, Croker DM, Walker GM, Goyanes A, Basit AW, Gaisford S (2018). Low temperature fused deposition modeling (FDM) 3D printing of thermolabile drugs. Int J Pharm.

[138] Ong JJ, Awad A, Martorana A, Gaisford S, Stoyanov E, Basit AW, Goyanes A (2020). 3D printed opioid medicines with alcohol-resistant and abuse-deterrent properties. Int J Pharm.

[139] Madzarevic M (2019). Vulovic, Sustersic, Djuris, Filipovic, Ibric, Optimization and Prediction of Ibuprofen Release from 3D DLP Printlets Using Artificial Neural Networks. Pharmaceutics.

[140] Wickström H, Koppolu R, Mäkilä E, Toivakka M, Sandler N (2020). Stencil Printing—A Novel Manufacturing Platform for Orodispersible Discs. Pharmaceutics.

[141] Rycerz K, Stepien KA, Czapiewska M, Arafat BT, Habashy R, Isreb A, Peak M, Alhnan MA (2019). Embedded 3D Printing of Novel Bespoke Soft Dosage Form Concept for Pediatrics. Pharmaceutics.

[142] Khaled SA, Burley JC, Alexander MR, Roberts CJ (2014). Desktop 3D printing of controlled release pharmaceutical bilayer tablets. Int J Pharm.

[143] G. Yõlmaz, R.O.J. Jongboom, H. Feil, W.E. Hennink, Encapsulation of sun¯ower oil in starch matrices via extrusion: effect of the interfacial properties and processing conditions on the formation of dispersed phase morphologies, Carbohydr. Polym. (2001) 8.

[144] Chang D, Abbas S, Hayat K, Xia S, Zhang X, Xie M, Kim JM (2010). Original article: Encapsulation of ascorbic acid in amorphous maltodextrin employing extrusion as affected by matrix/core ratio and water content: Encapsulation of ascorbic acid. Int J Food Sci Technol.

[145] B.H. Van Lengerich, Encapsulation of sensitive components into a matrix to obtaidiscrete shelf-stable particles., WO2001025414A1, n.d.

[146] Khor CM, Ng WK, Kanaujia P, Chan KP, Dong Y (2017). Hot-melt extrusion microencapsulation of quercetin for taste-masking. J Microencapsul.

[147] B.Y. Gajera, Investigating a Novel Hot Melt Extrusion-Based Drying Technique to Solidify an Amorphous Nanosuspension Using Design of Experiment Methodology, (n.d.) 13. 10.1208/s12249-018-1189-7 30280356

[148] Baumgartner R, Eitzlmayr A, Matsko N, Tetyczka C, Khinast J, Roblegg E (2014). Nano-extrusion: A promising tool for continuous manufacturing of solid nano-formulations. Int J Pharm.

[149] J.S. Koo, S.Y. Lee, O.K. Azad, M. Kim, S.J. Hwang, S. Nam, S. Kim, B.-J. Chae, W.-S. Kang, H.-J. Cho, extrusion and their therapeutic potentials for colon cancer, Int. J. Pharm. (2019) 8. 10.1016/j.ijpharm.2019.01.018 30665001

[150] Lee S, Nam S, Choi Y, Kim M, Koo J, Chae B-J, Kang W-S, Cho H-J (2017). Fabrication and Characterizations of Hot-Melt Extruded Nanocomposites Based on Zinc Sulfate Monohydrate and Soluplus. Appl Sci.

[151] Bhagurkar AM, Repka MA, Murthy SN (2017). A Novel Approach for the Development of a Nanostructured Lipid Carrier Formulation by Hot-Melt Extrusion Technology. J Pharm Sci.

[152] Patil H, Kulkarni V, Majumdar S, Repka MA (2014). Continuous manufacturing of solid lipid nanoparticles by hot melt extrusion. Int J Pharm.

[153] Guo M, Wei Y, Lee H, Maia J, Morrison E (2020). One-step extrusion of concentrated lidocaine lipid nanocarrier (LNC) dispersions. Int J Pharm.

[154] Bagde A, Patel K, Kutlehria S, Chowdhury N, Singh M (2019). Formulation of topical ibuprofen solid lipid nanoparticle (SLN) gel using hot melt extrusion technique (HME) and determining its anti-inflammatory strength, Drug Deliv. Transl Res.

[155] Blackshields CA, Crean AM (2018). Continuous powder feeding for pharmaceutical solid dosage form manufacture: a short review. Pharm Dev Technol.

[156] Ervasti T, Simonaho S-P, Ketolainen J, Forsberg P, Fransson M, Wikström H, Folestad S, Lakio S, Tajarobi P, Abrahmsén-Alami S (2015). Continuous manufacturing of extended release tablets via powder mixing and direct compression. Int J Pharm.

[157] Colón YM, Florian MA, Acevedo D, Méndez R, Romañach RJ (2014). Near Infrared Method Development for a Continuous Manufacturing Blending Process. J Pharm Innov.

[158] Quality Considerations for Continuous Manufacturing Guidance for Industry, (n.d.) 27.

